# Unveiling the Distinctive Brain Functional Dynamics Between Parkinson's Disease and Progressive Supranuclear Palsy

**DOI:** 10.1002/cns.71147

**Published:** 2026-09-04

**Authors:** Chenfei Ye, Jiahui Shi, Jiliang Fang, Tao Wu, Piu Chan, Jinghong Ma, Ting Ma

**Affiliations:** ^1^ School of Biomedical Engineering Harbin Institute of Technology (Shenzhen) Shenzhen China; ^2^ Department of Electronic and Information Engineering Harbin Institute of Technology (Shenzhen) Shenzhen China; ^3^ Department of Radiology Guang'anmen Hospital, China Academy of Chinese Medical Sciences Beijing China; ^4^ Department of Neurology, China National Clinical Research Center for Neurological Diseases Beijing Tiantan Hospital, Capital Medical University Beijing China; ^5^ Center for Movement Disorders, Department of Neurology Beijing Tiantan Hospital, Capital Medical University Beijing China; ^6^ Department of Neurology and Neurobiology Xuanwu Hospital Capital Medical University Beijing China; ^7^ Key Laboratory on Neurodegenerative Disorders of Ministry of Education Xuanwu Hospital Capital Medical University Beijing China; ^8^ Key Laboratory on Parkinson's Disease of Beijing Xuanwu Hospital Capital Medical University Beijing China; ^9^ Parkinson's Disease Center of Beijing Institute of Brain Disorders, Collaborative Innovation Center for Brain Disorders Xuanwu Hospital Capital Medical University Beijing China; ^10^ Department of Neurology Xuanwu Hospital of Capital Medical University Beijing China

**Keywords:** hidden Markov models, network dynamics, non‐motor impairment, Parkinson's disease, progressive supranuclear palsy

## Abstract

**Background:**

Dynamic analysis of resting‐state fMRI (rs‐fMRI) offers a novel approach to differentiate Parkinson's disease (PD) from progressive supranuclear palsy (PSP) by capturing temporal features of brain network activity, which may shed light on the mechanisms underlying non‐motor symptoms.

**Objectives:**

To characterize differences in brain dynamics between PD and PSP using dynamic brain metrics, evaluate their exploratory discriminative performance, and investigate associations with non‐motor symptoms.

**Methods:**

Sixty‐nine healthy controls, 82 PD patients, and 29 PSP patients underwent standardized clinical assessment and rs‐fMRI. Hidden Markov models extracted temporal features including fraction occurrence (FO), dwell time, and transition probability. These metrics were used for group comparisons, correlation analyses, and machine learning classification.

**Results:**

PD and PSP showed opposite trends in fraction occurrence of unimodal network‐dominant states. Compared to PD and controls, PSP exhibited prolonged duration in the dorsal attention and limbic network (DAN&LIM) state and increased occurrence in the dorsal attention and frontoparietal control network (DAN&FPCN) state. Reduced unimodal state occupancy correlated with cognitive decline in both groups, while increased DAN&LIM and unimodal persistence linked to worse mood and sleep disturbances in PSP. Machine learning with these metrics achieved moderate accuracy in differentiating PD from PSP.

**Conclusions:**

Temporal features of brain network dynamics may provide candidate imaging markers for distinguishing PD and PSP while offering mechanistic insights into non‐motor symptomatology. However, their diagnostic applicability requires validation in independent external multicenter cohorts.

## Graphical abstract

Using hidden Markov models (HMM) to analyze dynamic brain states from resting‐state fMRI, this study identifies opposing temporal dynamics in Parkinson's disease (PD) and progressive supranuclear palsy (PSP). PD patients show reduced fractional occupancy in unimodal networks, while PSP patients exhibit increased occupancy and prolonged duration in dorsal attention‐limbic states, correlating with cognitive decline and sleep disturbances. Machine learning based on HMM‐derived metrics achieves 89% accuracy in differentiating PD from PSP, providing robust biomarkers for precision diagnosis.

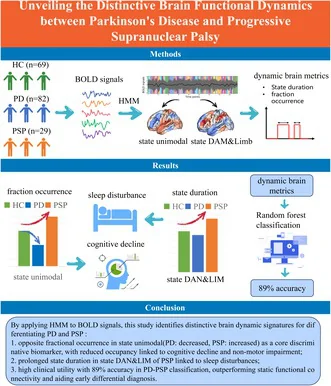

**Abbreviations**
AUC: area under the receiver operating characteristic curve
BOLD: blood oxygen level‐dependent
DAN: dorsal attention network
DMN: default mode network
ESS: Epworth Sleepiness Scale
FDR: false discovery rate
fMRI: functional magnetic resonance imaging
FO: fraction occurrence
FPCN: frontoparietal control network
GLMs: general linear models
HC: healthy controls
HMM: hidden Markov models
LEDD: Levodopa equivalent daily dose
LIM: Limbic network
MDS: Movement Disorder Society
MoCA: Montreal Cognitive Assessment
MRI: magnetic resonance imaging
NMSS: Non‐Motor Symptoms Scale
PD: Parkinson's disease
PSP: progressive supranuclear palsy
PSP‐CDS: Progressive Supranuclear Palsy Clinical Deficits Scale
PSPRS: Progressive Supranuclear Palsy Rating Scale
RBDQ‐HK: Hong Kong version of the REM Sleep Behavior Disorder Questionnaire
ROI: regions of interest
rs‐fMRI: resting‐state functional magnetic resonance imaging
SMN: somatomotor network
UPDRS: Unified Parkinson's Disease Rating Scale
VAN: ventral attention network
VIS: visual network

## 1 Introduction

Parkinson's disease (PD) and progressive supranuclear palsy (PSP) are neurodegenerative synuclein and tau‐related disorders that challenge clinical differentiation due to overlapping parkinsonian features, yet diverge profoundly in their molecular pathogenesis and network‐level consequences. PD arises from alpha‐synuclein aggregation and selective dopaminergic neuron loss in the substantia nigra pars compacta, disrupting nigrostriatal projections and basal ganglia‐thalamocortical loops, which manifests in bradykinesia, rigidity, and tremor with relatively preserved early cognition [1, 2]. In contrast, PSP is driven by 4R‐tau pathology, causing midbrain tegmental atrophy, basal ganglia tau inclusions, and frontal cortical involvement, leading to vertical gaze palsy, axial rigidity, and early executive dysfunction through impaired midbrain‐frontal connectivity [3, 4, 5]. These mechanistic distinctions—dopaminergic circuit vulnerability in PD versus tau‐mediated multisystem degeneration in PSP—underlie divergent clinical trajectories and treatment responses, but symptom overlap in early stages often leads to clinical misdiagnosis between PD and PSP [6, 7, 8].

To address the limitations of differential diagnosis, advanced neuroimaging techniques—especially resting‐state functional MRI (rs‐fMRI)—have been increasingly employed to map functional network alterations underpinning parkinsonian disorders. Rs‐fMRI allows for the non‐invasive assessment of spontaneous brain activity and is well‐suited to populations with motor disability [9]. Prior studies have demonstrated that, at a group level, PD is primarily associated with dysfunction within basal ganglia‐thalamo‐cortical loops and relative preservation of higher‐order cortical networks, whereas PSP is characterized by more pervasive disruption of frontoparietal and default mode networks [10, 11, 12]. However, traditional static connectivity measures overlook the dynamic and flexible nature of brain network architecture, thus failing to capture important transient changes that may more closely reflect the underlying disease mechanisms and offer increased sensitivity for differential diagnosis [13].

In recent years, dynamic functional connectivity approaches—most notably hidden Markov models (HMM)—have emerged as powerful tools to describe temporal variability in large‐scale brain networks. HMM can identify discrete recurrent brain states and quantify metrics such as fraction occurrence, dwell time, and transition probability, thereby revealing metastable neural dynamics and their pathological alteration [14, 15]. Dynamic brain state analysis has provided novel insights into the altered network transitions in parkinsonism, showing that specific metrics exhibit significant advantages in distinguishing patient groups and linking network dynamics to cognitive and non‐motor symptoms [16, 17]. Despite these advances, prior studies have predominantly focused on either PD or PSP in isolation, or in small samples, leaving a lack of research that directly and systematically compares brain state dynamics between PD and PSP under a unified analytic framework. This gap hinders our understanding of the distinct and overlapping network mechanisms underlying these diseases and the optimal development of imaging biomarkers for differential diagnosis.

To address these gaps, the present study retrospectively enrolled a relatively large cohort with a total of 180 subjects, including both PD and PSP patients who underwent an identical rs‐fMRI scanning protocol. By applying HMM‐based brain state analysis, we aimed to systematically characterize and compare dynamic functional connectivity patterns in PD and PSP, and to further relate these dynamic metrics to major non‐motor symptom domains. We hypothesized that PD and PSP would show distinct dynamic network profiles reflecting their disease‐related mechanisms, and that these differences might provide candidate imaging features for differentiating the two disorders and understanding disease heterogeneity.

## 2 Methods

### 2.1 Study Participants

A total of 180 individuals were enrolled, including 69 healthy controls (HC; 30 males, 39 females; mean age: 59.79 ± 9.39 years), 82 patients with Parkinson's disease (PD; 41 males, 41 females; mean age: 59.96 ± 8.6 years), and 29 patients with progressive supranuclear palsy (PSP; 19 males, 10 females; mean age: 67.24 ± 5.56 years). All participants meeting the study criteria were recruited from the Movement Disorders Clinic of Xuanwu Hospital, Capital Medical University. Patients with PD were diagnosed according to the Movement Disorder Society (MDS) clinical diagnostic criteria [18]. Patients with PSP were diagnosed by experienced movement disorder specialists according to the Movement Disorder Society criteria for progressive supranuclear palsy (MDS‐PSP criteria) [19]. PSP phenotypic variants were recorded according to the MDS‐PSP classification framework. Medication regimens before MRI acquisition were not standardized. Levodopa equivalent daily dose (LEDD) and the exact ON/OFF medication status at the time of scanning were not systematically recorded for the full cohort. Therefore, medication exposure could not be included as a covariate, and the effects of dopaminergic treatment could not be separated from disease‐related effects. This study was performed in accordance with the Declaration of Helsinki and was approved by the Institutional Review Board of Xuanwu Hospital, with approval number: 2022‐047. Written informed consent was provided by all participants before being enrolled in this study.

Comprehensive clinical assessments included the Movement Disorder Society‐Unified Parkinson's Disease Rating Scale (MDS‐UPDRS), Montreal Cognitive Assessment (MoCA), Non‐Motor Symptoms Scale (NMSS), Epworth Sleepiness Scale (ESS), Hong Kong version of the REM Sleep Behavior Disorder Questionnaire (RBDQ‐HK), and routine laboratory investigations. PSP‐specific clinical measures, including the Progressive Supranuclear Palsy Rating Scale (PSPRS) and Progressive Supranuclear Palsy Clinical Deficits Scale (PSP‐CDS), were collected when available. Disease duration was defined as the interval between the reported symptom onset and the date of MRI acquisition and was expressed in years. Detailed demographic and clinical characteristics are reported in Table 1.

**TABLE 1:** Demographics and clinical characteristics.

	HC	PD	PSP	*p* (HC vs. PD)	*p* (HC vs. PSP)	*p* (PD vs. PSP)
Age	59.79 ± 9.39	59.96 ± 8.6	67.24 ± 5.56	0.90	< 0.001^***^	< 0.001^***^
Sex	30 M/39F	41 M/41F	19 M/10F	0.525	0.077	0.221
Mean FD	0.17 ± 0.10	0.19 ± 0.11	0.18 ± 0.13	0.363	0.642	0.917
EDU (years)	11.85 ± 5.2	12.45 ± 4.45	11.0 ± 4.19	0.452	0.399	0.121
Course (years)	—	4.40 ± 3.41	3.77 ± 2.59	—	—	0.785
MDS‐UPDRS‐I	—	8.55 ± 5.81	12.2 ± 6.8	—	—	0.023
MDS‐UPDRS‐II	—	9.86 ± 6.22	16.4 ± 9.20	—	—	0.002
MDS‐UPDRS‐III	—	25.86 ± 12.76	31.16 ± 13.28	—	—	0.092
MoCA	25.03 ± 3.15	24.06 ± 3.49	19.6 ± 5.01	0.064	< 0.001^***^	< 0.001^***^
NMSS	11.93 ± 13.95	33.64 ± 33.48	44.25 ± 32.11	< 0.001^***^	< 0.001^***^	0.213
RBDQ_HK	9.23 ± 6.63	20.49 ± 16.28	12.54 ± 15.09	< 0.001^***^	0.339	0.042
ESS	4.46 ± 3.39	7.25 ± 4.99	5.92 ± 5.48	0.014	0.237	0.799

*Note:* All data are presented in mean ± standard deviation mode, and significant comparisons with FDR corrected *p* < 0.05 are bolded. EDU represents participants' years of formal education, measured in years; Course represents participants' years of disease duration, measured in years.

Abbreviations: ESS, Epworth Sleepiness Scale; HC, Healthy Control; MDS‐UPDRS‐I, MDS‐UPDRS‐II, MDS‐UPDRS‐III, Movement Disorder Society‐Sponsored Revision of the Unified Parkinson's Disease Rating Scale, part I, II, III; Mean FD, mean framewise displacement; MoCA, Montreal Cognitive Assessment; NMSS, Non‐Motor Symptoms Scale; PD, patients with Parkinson's disease; PSP, patients with progressive supranuclear palsy; RBDQ‐HK, Hong Kong version of the REM Sleep Behavior Disorder Questionnaire.

*** *p* < 0.001.

### 2.2 Data Acquisition and Preprocessing

All imaging was performed on a 3T Siemens Magnetom Skyra scanner with harmonized protocols across participants. The T1‐weighted structural imaging preprocessing was first performed according to FreeSurfer *recon‐all* pipeline. Then the rs‐fMRI preprocessing included temporal realignment, image registration and motion correction, spatial normalization, spatial smoothing, and frequency‐domain filtering, using the fMRIPrep software [20]. During quality control, participants with head motion displacements greater than 2.5 mm during fMRI scanning were excluded from subsequent analysis. Next, the preprocessed fMRI images were mapped to the Schaefer 400‐parcel brain functional atlas [21], with the resulting brain regions serving as the network nodes for subsequent individual brain network construction. Full scanning protocols are described in the Supporting Information.

### 2.3 Characterization of Brain States

**FIGURE 1:**
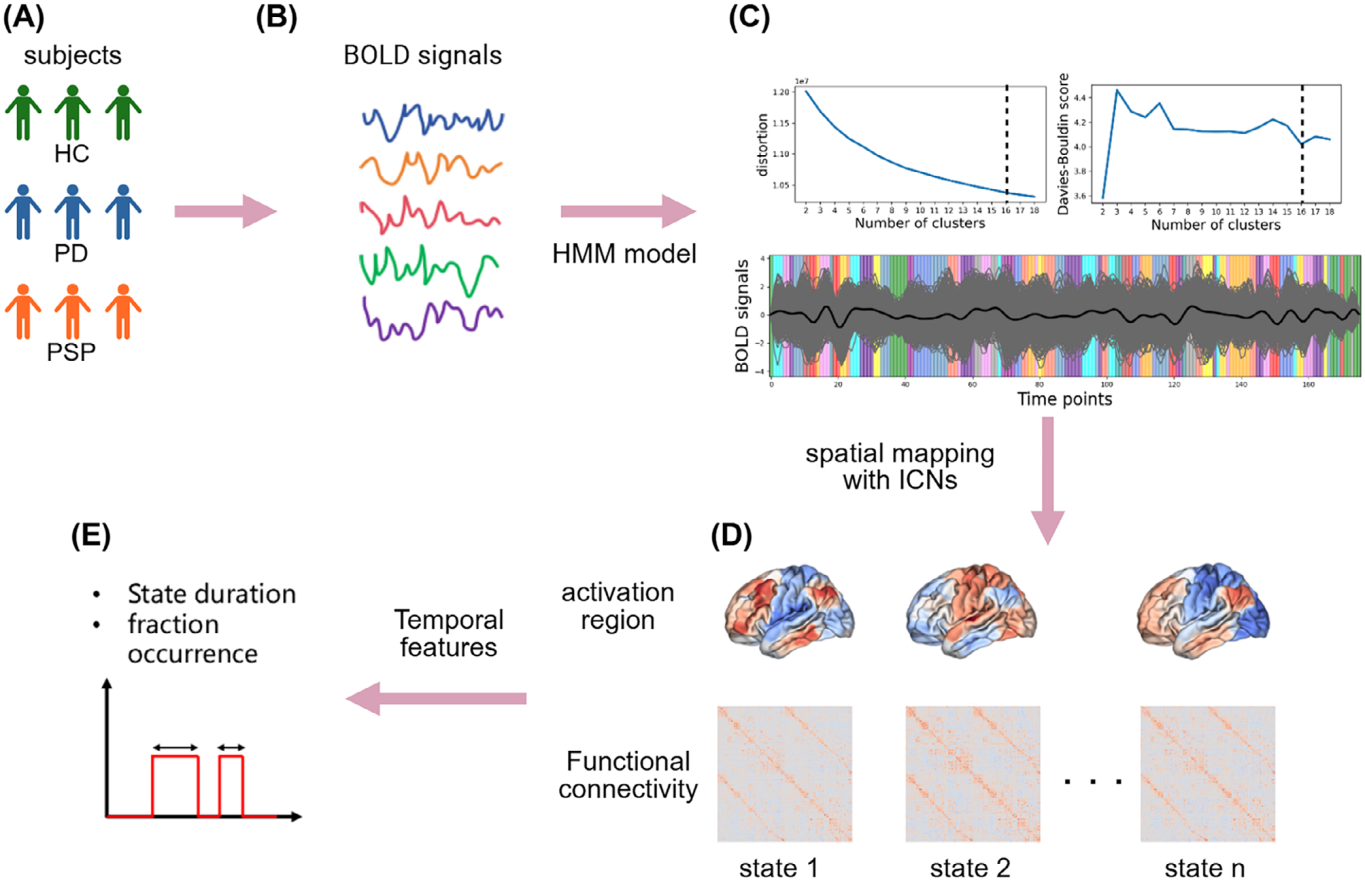
Schematic flowchart of the study methodology. (A) The study included 180 subjects: 69 healthy controls, 82 patients with PD, and 29 patients with PSP. (B) Whole‐brain parcellation was performed using 400 cortical regions, and brain dynamics were derived from each participant's rs‐fMRI data. (C) The hidden Markov model (HMM) was used to identify dynamic brain states, with cluster numbers evaluated by distortion and Davies‐Bouldin index. Sixteen clusters were selected as optimal, and each time point of each subject was assigned to one state accordingly. (D) Spatial mapping with intrinsic connectivity networks was used to analyze activity regions and their associated dynamic functional connectivity matrices under different states. (E) Temporal features of each state, including frequency of occurrence and mean duration, were calculated.

Traditional sliding window approaches for dynamic connectivity are limited by arbitrary parameter choices and motion sensitivity [22, 23, 24, 25]. To overcome these challenges, we applied hidden Markov modeling (HMM) to the preprocessed rs‐fMRI time series, a framework which infers discrete, mutually exclusive brain states and their temporal transitions [16, 26] (see Figure 1). The HMM was implemented using the *hmmlearn* Python package (https://github.com/hmmlearn/hmmlearn). Specifically, we used a Gaussian‐emission HMM with diagonal covariance structure, as the input data consisted of continuous ROI‐wise BOLD signals and this model provides a parsimonious and interpretable representation of latent brain states while reducing over‐parameterization compared with more complex emission models. Before HMM fitting, each ROI‐wise BOLD time series was *z*‐scored across time separately within each participant, resulting in zero mean and unit variance for each ROI. No additional standardization was performed across parcels or across participants. Each participant‐specific time‐by‐ROI matrix was then concatenated sequentially along the temporal dimension to form a single group‐level input matrix. Participant sequence lengths were not explicitly supplied during HMM fitting or Viterbi decoding; therefore, the concatenated data were treated as a single continuous sequence at these stages. After decoding, the inferred state labels were separated according to participant identifiers, and fractional occupancy, mean dwell time, and transition probabilities were calculated separately within each participant. Consequently, transitions across participant boundaries were not included in the calculation of subject‐level dynamic metrics.

For the full cohort, candidate HMMs with model orders ranging from *k* = 2 to *k* = 18 were fitted to the concatenated ROI‐wise BOLD signal matrix. For each model order, the HMM was fitted using 20 random initializations, and the solution with the highest log‐likelihood was retained. Model parameters were estimated using the expectation–maximization algorithm implemented in *hmmlearn*, with a maximum of 1000 iterations and a convergence tolerance of 1 × 10^−6^. The most likely latent state sequence was then obtained using Viterbi decoding. The 16‐state solution was selected because it provided a favorable balance between model fit, cluster separation, parsimony, and neurobiological interpretability, as indicated by the distortion index and Davies–Bouldin index. Spatial profiles of each HMM‐identified state were aligned to canonical cortical networks as defined by Yeo et al. [27] to facilitate neuroanatomical interpretation. The correlations between state spatial maps and canonical networks are depicted in Figure S1.

Three principal dynamic metrics were computed: fraction occurrence (the proportion of time spent in each state, reflecting its temporal prevalence and stability), mean state duration (average consecutive time‐points spent within a state, i.e., state lifetime), and transition probability (likelihood of switching from one state to another, reflecting the temporal flexibility of network configurations). These metrics were carried forward to group comparisons and to explore the neural underpinnings of clinical manifestations in PD and PSP.

### 2.4 Statistical Analyses

Group differences in fraction occurrence and mean state duration were examined using general linear models with group as the main factor and age, sex, and mean framewise displacement (mean FD) included as covariates. Group differences were assessed using covariate‐adjusted GLMs, and pairwise contrasts were estimated within the same model. Post hoc pairwise comparisons between groups were subsequently performed. Differences in transition probabilities were assessed using Mann–Whitney *U* tests due to non‐normal distributions.

False discovery rate (FDR) correction was applied within predefined statistical families. For fraction occurrence and mean dwell time, FDR correction was performed across the 16 HMM states separately for each pairwise group contrast. Omnibus group‐effect tests were also corrected across the 16 states within each metric. For transition probabilities, FDR correction was applied across the full 16 × 16 transition matrix separately for each pairwise group contrast. Results not surviving FDR correction were considered exploratory.

Associations between brain dynamic metrics and clinical features were examined using partial correlation analyses controlling for age and sex, and mean framewise displacement. Given the limited PSP sample size, PSP‐specific partial correlations were further evaluated using bootstrap resampling with 10,000 iterations to assess the stability of correlation coefficients and to estimate bootstrap‐based 95% confidence intervals. Categorical variables were compared using chi‐square tests. Effect sizes were reported with 95% confidence intervals where appropriate. For PSP‐specific analyses, partial correlations were examined between each of the two PSP‐specific clinical scales (PSPRS and PSP‐CDS) and all 16 fraction occurrence and 16 mean dwell time metrics. Analyses were performed using complete cases with available clinical scale scores, HMM‐derived metrics, age, sex, and mean framewise displacement. The exact sample size was determined separately for each clinical scale and dynamic metric. Because the required HMM metrics and covariates were complete among participants with available PSP‐specific scale scores, 24 PSP patients were included in all PSPRS analyses and 24 PSP patients were included in all PSP‐CDS analyses. FDR correction was applied separately within each scale by metric family.

Given the unequal sample sizes across groups, sensitivity power analyses were performed for key group comparisons and PSP‐specific correlation analyses. For group comparisons involving PSP, the minimum detectable Cohen's *d* was estimated for PSP vs. PD and PSP vs. HC comparisons at *α* = 0.05 and 80% power. Positive Cohen's *d* values indicate higher values in the first‐listed group relative to the second‐listed group. For PSP‐specific correlation analyses, the minimum detectable correlation coefficient was estimated using Fisher's *z* transformation.

### 2.5 Discriminative Power of Brain State Features

To assess exploratory diagnostic potential, dynamic HMM features and static functional connectivity (FC) features were compared using the same nested stratified cross‐validation framework. For the dynamic HMM model, candidate predictors included fraction occurrence and mean state duration across the 16 HMM states, together with transition probability features. Feature selection was performed within the cross‐validation pipeline rather than before model training. For the static FC benchmark, Schaefer400 regional BOLD time series were used to compute Pearson correlation coefficients between all pairs of parcel‐wise time series. Because the exported time‐series matrices contained parcels as rows and fMRI time points as columns, matrices were transposed before FC computation. Correlation coefficients were Fisher *z*‐transformed, and upper triangular off‐diagonal elements were vectorized, yielding 79,800 FC edge features per participant. A nested stratified cross‐validation framework was used: the inner loop performed feature selection and random forest hyperparameter tuning, whereas the outer loop estimated held‐out classification performance. Class imbalance between PD and PSP was addressed using class‐weighted random forest classifiers. Classification performance was evaluated using accuracy, balanced accuracy, sensitivity, specificity, and area under the receiver operating characteristic curve (AUC). Feature contribution was assessed by feature‐selection frequency across outer folds.

### 2.6 Sensitivity Analyses

Robustness of HMM‐derived states was assessed across a range of clustering numbers (*k* = 12–17) and parcellation atlases (Schaefer‐400, Schaefer‐200). Topographical consistency was evaluated using spatial correlation metrics and a null model with rotational permutations that preserved spatial autocorrelation [28, 29]. Metrics included Pearson and Spearman correlation coefficients evaluated over 10,000 permutations. To evaluate the robustness of the primary findings after reducing the between‐group age imbalance, we constructed an age‐balanced subset using manual age‐based trimming. All 29 participants with PSP were retained. Participants in the HC and PD groups were ranked by age in ascending order, and the youngest individuals were sequentially excluded from each group. A total of 26 HC participants and 38 participants with PD were excluded, resulting in an age‐balanced subset comprising 43 HC participants, 44 participants with PD, and 29 participants with PSP (total *n* = 116). Participant selection was based exclusively on age and was performed without reference to HMM‐derived metrics or statistical outcomes. No random sampling, replacement, or nearest‐neighbor matching was used. Because residual age differences remained, this sample is referred to as an age‐balanced subset rather than an age‐matched sample.

The HMM was refitted independently in the age‐balanced subset, and candidate model orders ranging from *k* = 2 to *k* = 20 were evaluated in this reduced sample. The original full‐cohort *k* = 16 HMM was not directly applied to the age‐balanced subset. Because the two HMMs were fitted using different participant samples, model order, state configuration, and numerical state labels were solution specific. Corresponding states were therefore identified according to their spatial network profiles rather than their numerical identifiers. Based on the model‐quality indices, parsimony, and spatial interpretability within the age‐balanced subset, the *k* = 19 solution was selected for detailed group comparisons. Because state numbers are solution specific, states were compared across solutions according to their spatial network profiles rather than their numerical identifiers; a state‐numbering key is provided in Table S7. Comparisons between PD and PSP may be influenced not only by disease‐specific mechanisms but also by disparities in disease stage, clinical severity, disease duration and overall neurodegenerative burden. To account for such confounding effects, the GLMs were refitted in participants with PD and PSP by separately adding disease duration, MDS‐UPDRS‐III and MDS‐UPDRS‐I as an additional covariate, while retaining age, sex, and mean FD. Analyses were based on complete cases, and FDR correction was applied across the 16 states separately within each dynamic‐metric family and model.

## 3 Results

### 3.1 Demographic and Clinical Characteristics

A total of 29 patients with PSP, 82 patients with PD, and 69 healthy controls (HC) were included in the analysis. Among PSP patients, Richardson syndrome (PSP‐RS) was the predominant phenotype, accounting for the majority of classified cases, while smaller numbers of patients presented with alternative phenotypes including PSP‐parkinsonism (PSP‐P), PSP‐corticobasal syndrome (PSP‐CBS), PSP‐progressive gait freezing (PSP‐PFG), and other variants. A subset of patients could not be confidently assigned to a specific phenotype based on the available clinical information. Age was comparable between PD patients (59.96 ± 8.60 years) and healthy controls (HC, 59.79 ± 9.39 years, *p* = 0.90), whereas PSP patients were notably older (67.24 ± 5.56 years) than both PD and HC (both *p* < 0.001). Although the PSP group showed a numerically higher proportion of males compared with the HC group, the difference did not reach statistical significance (*p* = 0.077). No significant sex differences were observed between PD and HC (*p* = 0.525) or between PD and PSP (*p* = 0.221). Years of education (EDU) did not differ significantly across the three groups (HC: 11.85 ± 5.2; PD: 12.45 ± 4.45; PSP: 11.0 ± 4.19; one‐way ANOVA: F = 1.023, *p* = 0.361). Both PD and PSP groups exhibited higher non‐motor symptom (NMSS) scores relative to controls (33.64 ± 33.48 and 44.25 ± 32.11 vs. 11.93 ± 13.95; both *p* < 0.001), while PSP patients had numerically higher NMSS scores than PD, though this was not statistically significant (*p* = 0.213). Compared to PD patients, PSP patients showed greater impairment on MDS‐UPDRS‐I (12.2 ± 6.8 vs. 8.55 ± 5.81, *p* = 0.023) and MDS‐UPDRS‐II (16.4 ± 9.20 vs. 9.86 ± 6.22, *p* = 0.002), and exhibited more severe cognitive decline as reflected by MoCA (19.6 ± 5.01 vs. 24.06 ± 3.49, *p* < 0.001); additionally, both PD and PSP had lower MoCA scores than controls (25.03 ± 3.15), with the difference being significant for PSP vs. HC (*p* < 0.001) and not for PD vs. HC (*p* = 0.064). For REM sleep behavior and daytime sleepiness, PSP patients exhibited intermediate RBDQ‐HK scores (12.54 ± 15.09 vs. 9.23 ± 6.63 for HC, *p* = 0.339; vs. 20.49 ± 16.28 for PD, *p* = 0.042) and ESS scores (5.92 ± 5.48 vs. 4.46 ± 3.39 for HC, *p* = 0.237; vs. 7.25 ± 4.99 for PD, *p* = 0.799), with significant differences observed between PD and PSP in RBDQ‐HK scores (*p* = 0.042). MDS‐UPDRS‐III scores were marginally higher in PSP than PD (31.16 ± 13.28 vs. 25.86 ± 12.76, *p* = 0.092), but this difference was not statistically significant. Detailed statistics are summarized in Table 1.

### 3.2 Characterization of Brain States

**FIGURE 2:**
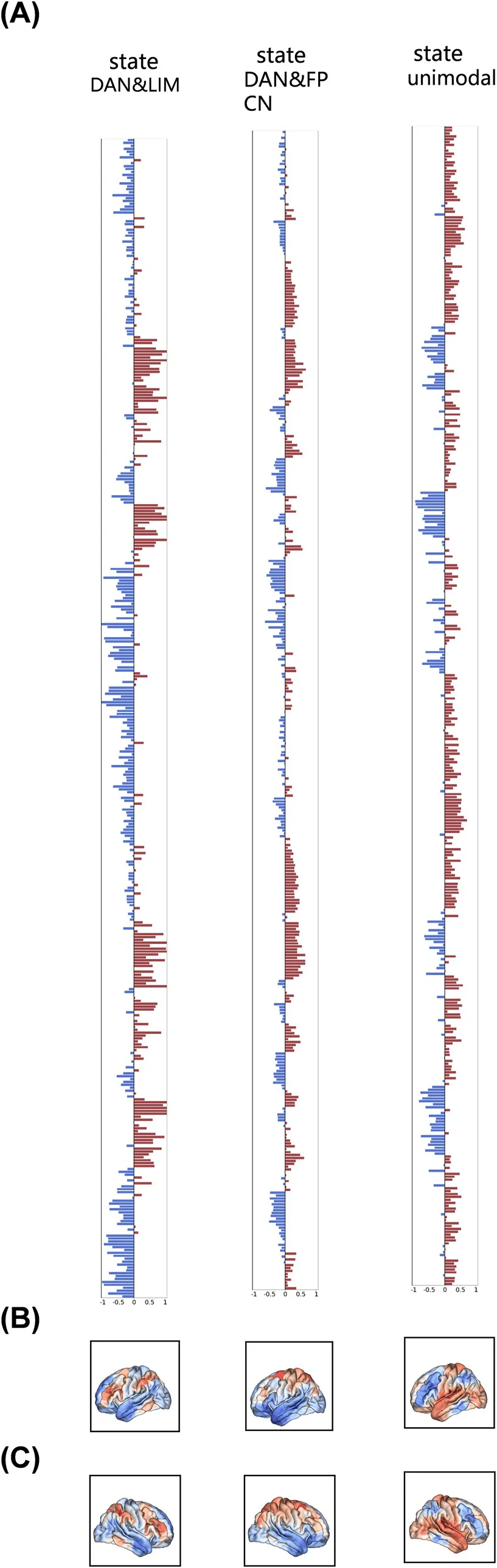
Representative transient brain states identified across all participants. (A) Cluster centers of selected HMM derived states using Schaefer400 parcels, including the DAN&LIM state, DAN&FPCN state, and unimodal state. (B) Visualization of cluster centers on the right lateral brain surface. (C) Visualization of cluster centers on the left lateral brain surface.

Sixteen distinct brain states were identified from the HMM analysis of rs‐fMRI data, with each fMRI time point assigned to a specific state based on the Schaefer 400‐region atlas. Representative states most relevant to the main group differences are shown in Figure 2, and the full set of 16 states is provided in Figure S2. The fraction occurrence of the unimodal state remained consistent across different cluster numbers (*k* = 12–17), as shown in Figure S3. Among these states, four exhibited predominant synchronization within the default mode network (DMN), while two states displayed synchronized activity in the visual network (VIS), and another two in the somatomotor network (SMN). One global synchronization state exhibited widespread concordance across cortical parcels. Two additional states were regionally focused, one involving the limbic cortex and the other the dorsal attention network (DAN). Additional states were characterized by co‐activation across network boundaries—including states combining DAN and limbic cortex, DAN and frontoparietal control network (FPCN), SMN and VIS (termed “unimodal state”), and conjunctions of DMN, ventral attention network (VAN), and FPCN (“transmodal state”). One state did not align with standard canonical network definitions.

### 3.3 Alterations in Modular Brain Dynamics

**FIGURE 3:**
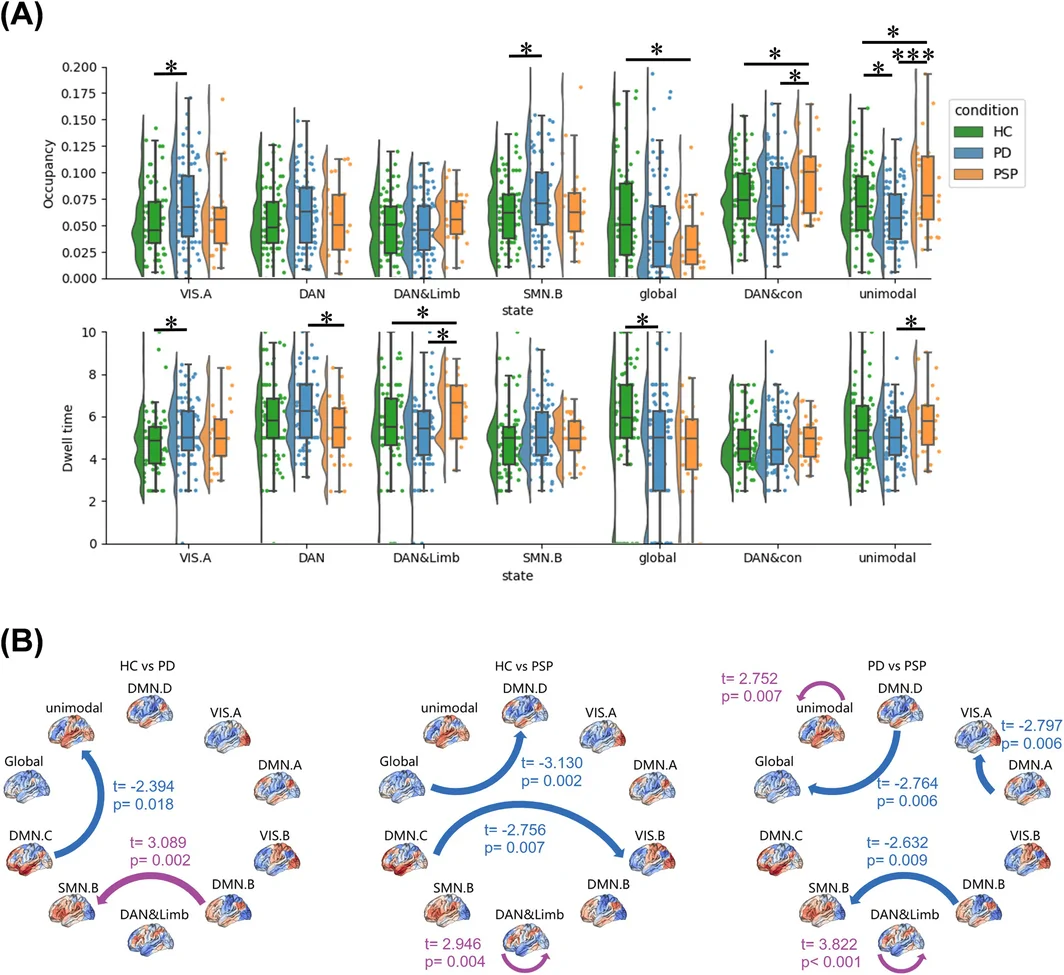
Brain dynamics in PD, PSP, and healthy controls. (A) Group comparison of frequency of occurrence and mean duration for each state. State duration is defined as the average time spent in a particular state during each fMRI scan. Significance levels: *p < 0.05, **p < 0.01 (FDR‐corrected). Healthy controls are shown in green; PD patients in blue; and PSP patients in orange. (B) Transition probabilities for ten out of sixteen HMM‐inferred states: Group‐averaged transition probability matrices are shown for each group. Each brain map represents a state, and arrows indicate probabilistic transitions between states. Arrow color corresponds to group‐averaged transition probability (red for increased probability, blue for decreased probability).

We comprehensively compared fraction occurrence and mean state duration across the 16 HMM‐derived brain states using general linear models adjusted for age, sex, and mean FD (Figure 3). The most robust finding involved fraction occurrence of the unimodal state (state 15). In the omnibus GLMs, state 15 showed a significant overall group effect that survived FDR correction across the 16 states (*F* = 7.57, *p* = 0.0007, pFDR = 0.011). Raw group means showed an opposing pattern, with lower unimodal fraction occurrence in PD (0.059 ± 0.029) and higher occupancy in PSP (0.088 ± 0.037) relative to HC (0.072 ± 0.032). Planned post hoc contrasts demonstrated that the direct PSP vs. PD difference survived FDR correction across the 16 states within that pairwise comparison (*β* = 0.028, 95% CI [0.013, 0.042], *p* = 0.0003, pFDR = 0.004). Comparisons of PD vs. HC and PSP vs. HC showed directionally consistent nominal effects but did not survive within‐comparison FDR correction. These results identify unimodal fraction occurrence as the primary corrected dynamic marker differentiating PD from PSP. Full fraction occurrence statistics for all 16 HMM states, including omnibus and pairwise comparisons, are provided in Table S1. Corresponding mean dwell time statistics for all 16 HMM states are summarized in Table S2.

PSP patients also showed supportive increases in fraction occurrence of the DAN&FPCN state (state 14). Raw group means were 0.078 ± 0.031 in HC, 0.077 ± 0.033 in PD, and 0.096 ± 0.036 in PSP. In covariate‐adjusted post hoc analyses, PSP showed higher state 14 occupancy than PD (*β* = 0.020, 95% CI [0.006, 0.034], *p* = 0.006, pFDR = 0.051) and HC (*β* = 0.018, 95% CI [0.003, 0.033], *p* = 0.017, pFDR = 0.164). The omnibus group effect was nominal (*F* = 3.95, *p* = 0.021, pFDR = 0.108). Therefore, the DAN&FPCN result was retained as a secondary supportive finding rather than a primary FDR‐corrected effect.

For mean state duration, PSP patients showed numerically prolonged duration in the DAN&LIM state (state 9; HC: 5.51 ± 2.00, PD: 5.34 ± 2.01, PSP: 6.40 ± 1.42). Planned comparisons showed nominally longer state 9 duration in PSP than PD (*β* = 0.946, 95% CI [0.090, 1.803], *p* = 0.031, pFDR = 0.129) and a similar direction compared with HC (*β* = 0.834, 95% CI [−0.046, 1.714], *p* = 0.063, pFDR = 0.352). Because this effect was attenuated after adjustment for age, sex, and mean FD and did not survive FDR correction, it was interpreted as an exploratory supportive finding indicating possible attentional‐limbic persistence in PSP.

Brain state transition probability matrices revealed that most transitions reflected recurrent dwelling within the same state, with self‐transition probabilities exceeding 82% (Figure 3B). Transition probability analyses were conducted across the complete transition matrix, including self‐transitions. Several transitions showed nominal group differences prior to correction, including increased transitions from DMN.B to SMN.B in PD patients relative to HC and PSP, as well as reduced transitions from the global state to DMN.D in PSP patients relative to both PD and HC. However, these findings did not survive FDR correction across the full transition matrix and should be interpreted cautiously as exploratory observations. Complete transition‐level statistics, including raw and FDR‐corrected *p* values, are provided in the Supporting Information.

In disease duration sensitivity analyses, the unimodal‐state fraction occurrence difference between PSP and PD remained significant after additional adjustment for disease duration (*β* = 0.0269, 95% CI [0.0107, 0.0431], *p* = 0.0014, qFDR = 0.0226). The effect remained similar in magnitude and direction after adjustment for MDS‐UPDRS‐III, although it did not survive FDR correction (*β* = 0.0241, *p* = 0.0037, qFDR = 0.0584). Exploratory adjustment for MDS‐UPDRS‐I attenuated the effect (*β* = 0.0186, *p* = 0.0168, qFDR = 0.2691). Full results and complete‐case sample sizes are provided in Table S6.

### 3.4 Association Between Brain Dynamics and Clinical Measures

**FIGURE 4:**
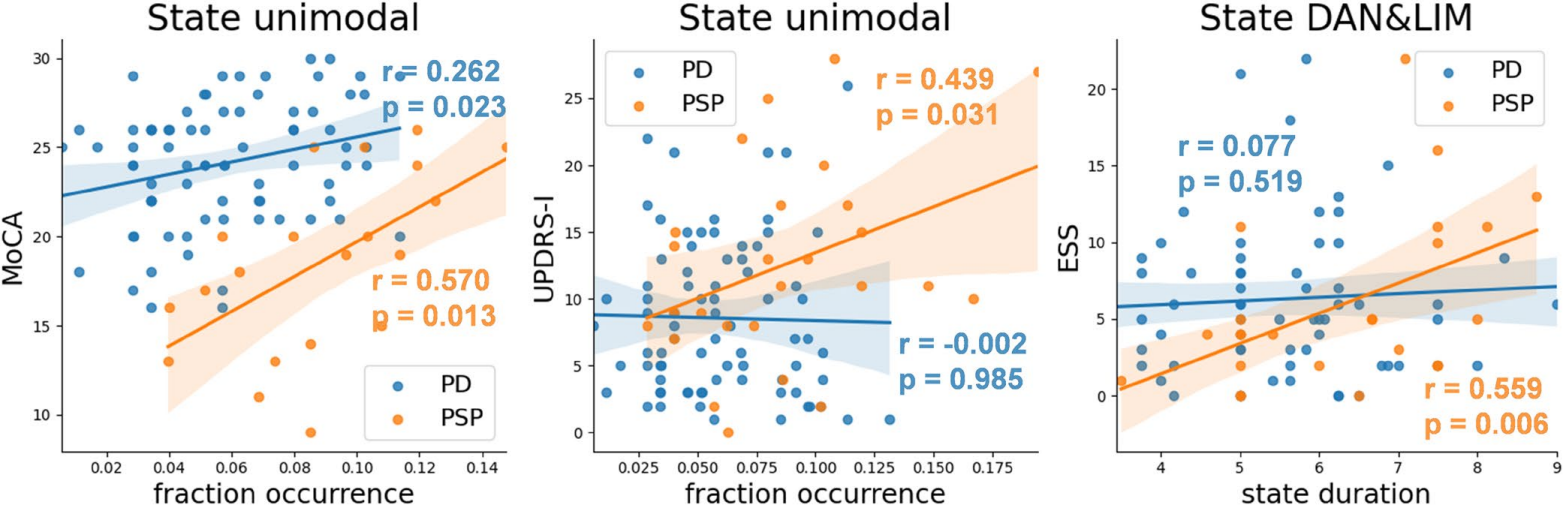
Correlations between dynamic brain state features and non‐motor symptoms in PD and PSP. Associations of state dynamics with clinical symptoms are shown for PD and PSP patients. Increased frequency of occurrence in the unimodal state is correlated with improved MoCA scores in both groups. In PSP, higher fraction occurrence in the unimodal state is associated with more severe non‐motor symptoms, a pattern not observed in PD. Increased duration in the DAN&LIM state is correlated with greater daytime sleepiness in PSP, but not in PD. PD patients are shown in blue; PSP patients in orange.

**FIGURE 5:**
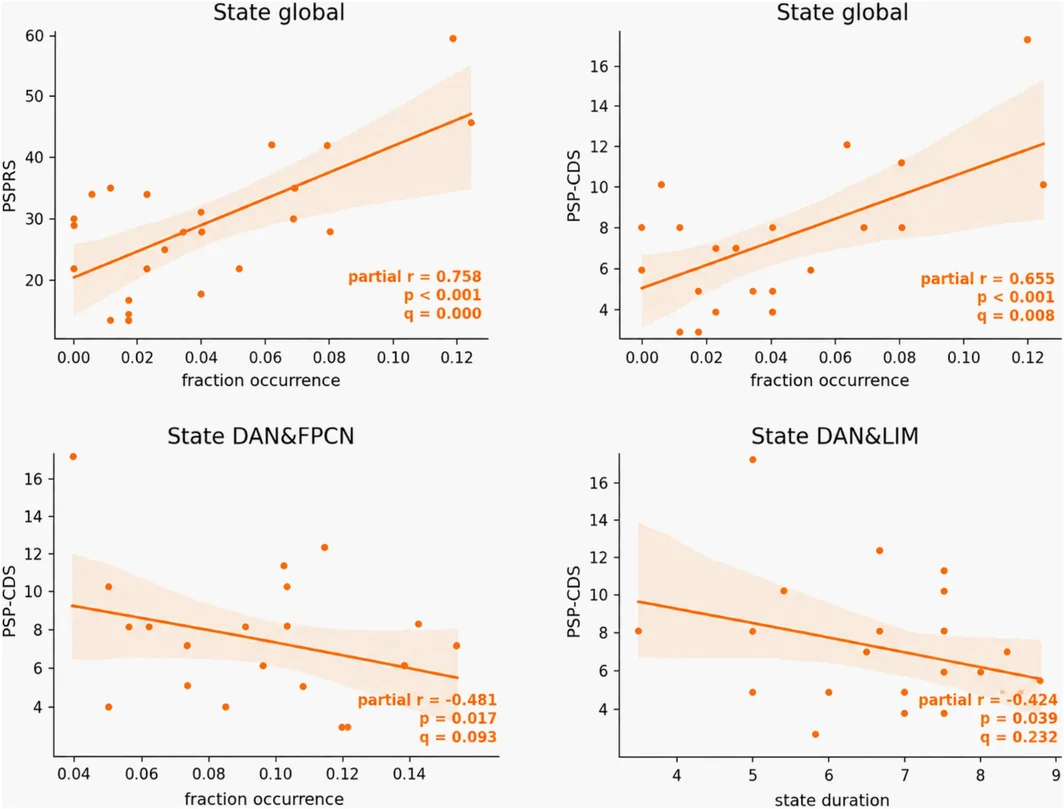
Global synchronization state fraction occurrence correlates with disease‐specific severity in progressive supranuclear palsy. Correlations between dynamic brain state features and clinical measures. PSP‐specific severity scales were associated with fraction occurrence of the global state: PSPRS and PSP‐CDS showed positive covariate‐adjusted partial correlations in PSP patients. PSPRS, Progressive Supranuclear Palsy Rating Scale; PSP‐CDS, Progressive Supranuclear Palsy Clinical Deficits Scale.

Partial correlation analyses were performed for brain dynamic metrics that differed significantly between groups. In PSP patients, higher fraction occurrence of the unimodal state was positively associated with MoCA scores (*r* = 0.57, 95% CI [0.14, 0.82], *p* = 0.01; bootstrap 95% CI [0.26, 0.82], bootstrap *p* = 0.004), indicating that greater occupancy was associated with better cognitive performance. Separately, higher unimodal‐state fraction occurrence was positively associated with MDS‐UPDRS‐I scores (*r* = 0.44, 95% CI [0.062 to 0.745], *p* = 0.03; bootstrap *p* = 0.01), indicating an association with greater non‐motor symptom burden. These two associations were interpreted separately because higher MoCA and higher MDS‐UPDRS‐I scores represent opposite clinical directions. In PD patients, fraction occurrence of the unimodal state was positively correlated with MoCA scores (*r* = 0.27, *p* = 0.02), but not with MDS‐UPDRS‐I scores (*r* = −0.002, *p* = 0.98) (Figure 4A). No significant relationships were observed between dynamic brain state features and motor disability as measured by MDS‐UPDRS‐III in either group. In PSP patients, increased mean state duration in the DAN&LIM state was strongly correlated with ESS scores (*r* = 0.56, 95% CI [0.18, 0.79], *p* = 0.006; bootstrap 95% CI [0.25, 0.80], bootstrap *p* = 0.0056), suggesting that prolonged occupancy of this state is linked to greater sleep disturbances. PSP‐specific analyses further revealed robust positive associations between fraction occurrence of the global state and PSP‐specific disease severity. PSPRS correlated strongly with global state fraction occurrence (*n* = 24; partial *r* = 0.758, *p* = 1.8 × 10–5, q = 0.0003; bootstrap 95% CI, 0.487 to 0.923; bootstrap *p* = 0.0006). PSP‐CDS showed a similar association with global state fraction occurrence (*n* = 24; partial *r* = 0.655, *p* = 0.0005, q = 0.008; bootstrap 95% CI, 0.285 to 0.880; bootstrap *p* = 0.0042) (Figure 5). Complete PSP‐specific correlation results across all HMM metrics are shown in Figure S4. Bootstrap analyses yielded confidence intervals consistent with the parametric estimates, supporting the robustness of PSP‐specific associations despite the relatively small sample size. Detailed PSP‐specific partial correlations, including bootstrap 95% CIs and FDR‐corrected q‐values, are presented in Table S3. Sensitivity power analyses indicated that, given the available sample sizes, the study had 80% power to detect moderate‐to‐large group differences involving PSP (minimum detectable Cohen's *d* ≈ 0.61–0.62) and PSP‐specific correlations of approximately *r* = 0.49 or greater at *α* = 0.05. Complete targeted clinical correlation results for the three HMM‐derived metrics across clinical measures in PD and PSP, including exact sample sizes, 95% confidence intervals, raw *p* values, and within‐family FDR‐adjusted *q* values, are provided in Table S8. Complete PSP‐specific correlations of PSPRS and PSP‐CDS with all 16 fraction occurrence and 16 mean dwell time metrics are provided in Table S9.

### 3.5 Classification Performance: Differentiation of PD and PSP

**FIGURE 6:**
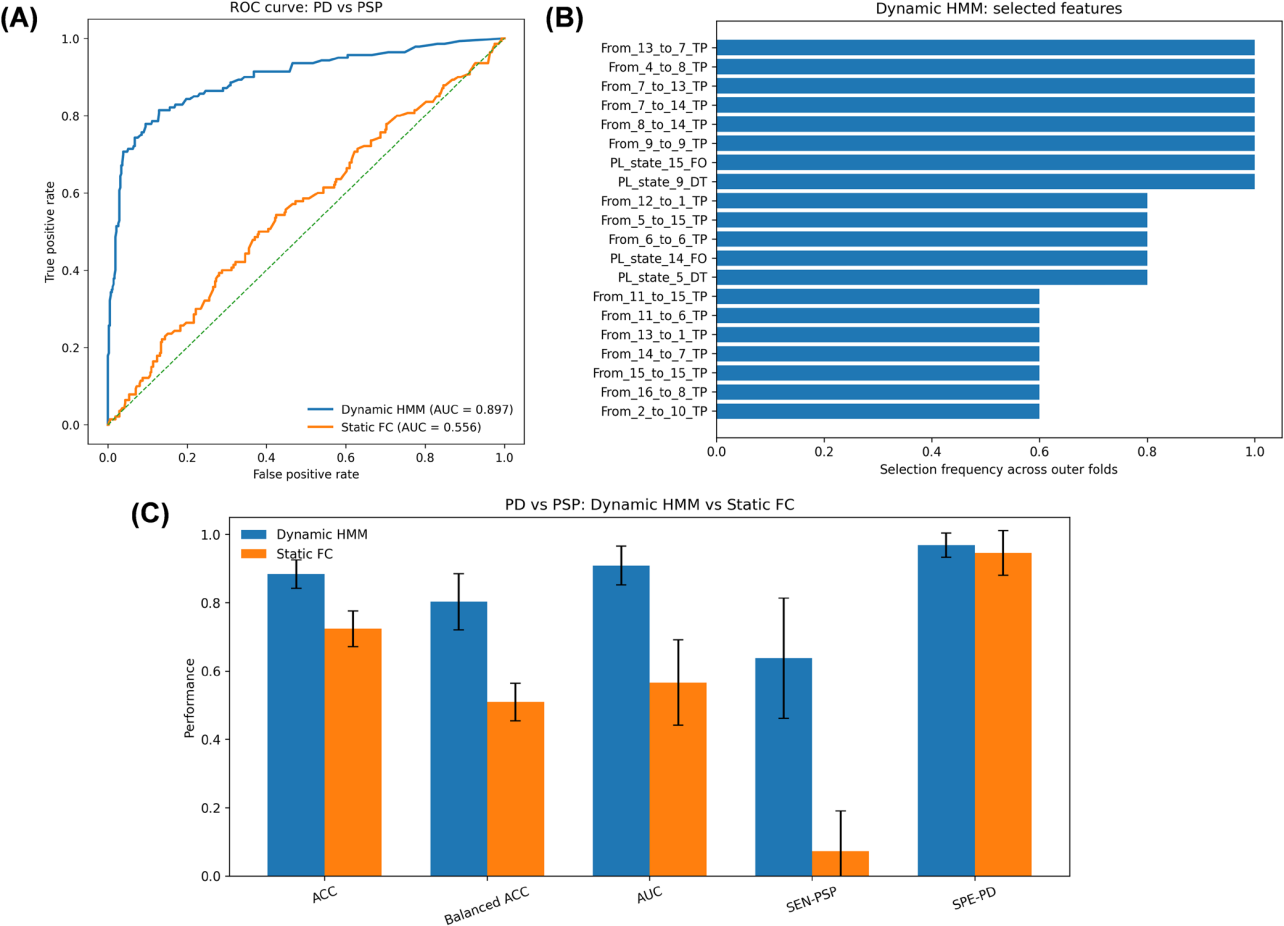
Exploratory machine‐learning classification of PD and PSP. (A) ROC curves for dynamic HMM and static FC models. (B) Dynamic‐feature selection frequency across outer folds. Key selected features included state 15 fraction occurrence, state 9 mean duration, and state 14 fraction occurrence. (C) Classification performance under the same nested stratified cross‐validation framework.

To address class imbalance and potential information leakage, the PD‐vs‐PSP classification analysis was re‐performed using a nested stratified cross‐validation framework. Dynamic HMM features and static FC features were evaluated under the same class‐weighted random forest pipeline (Table 2; Figure 6). The dynamic HMM feature set showed stronger classification performance than static FC, with accuracy of 0.884 ± 0.042, balanced accuracy of 0.803 ± 0.082, and AUC of 0.908 ± 0.057. Sensitivity for PSP was 0.637 ± 0.176, and specificity for PD was 0.969 ± 0.035. This class‐specific imbalance indicates that, despite the high overall AUC, the model was less effective in identifying PSP cases than in correctly classifying PD cases. In contrast, the static FC model showed weaker performance, with accuracy of 0.724 ± 0.052, balanced accuracy of 0.509 ± 0.055, and AUC of 0.566 ± 0.125. Sensitivity for PSP and specificity for PD were 0.072 ± 0.118 and 0.946 ± 0.065, respectively. Feature‐selection frequency analysis further showed that several key HMM‐derived features were consistently selected across outer folds. Notably, unimodal fraction occurrence and DAN&LIM mean duration were selected in all outer folds, whereas DAN&FPCN fraction occurrence was selected in most folds. These results suggest that the dynamic classifier relied partly on the same biologically interpretable features identified in the group‐level analyses. Because the classification analysis was internally validated but not tested in an independent external cohort, these findings should be interpreted as exploratory evidence for diagnostic potential rather than as clinically validated performance.

**TABLE 2:** Classification accuracy based on combined temporal dynamic metrics and FC.

Feature set	Accuracy	Balanced accuracy	AUC	Sensitivity (PSP)	Specificity (PD)
Dynamic HMM	0.884 ± 0.042	0.803 ± 0.082	0.908 ± 0.057	0.637 ± 0.176	0.969 ± 0.035
Static FC	0.724 ± 0.052	0.509 ± 0.055	0.566 ± 0.125	0.072 ± 0.118	0.946 ± 0.065

*Note:* Values are reported as mean ± standard deviation across outer folds of nested stratified cross‐validation. Sensitivity refers to PSP detection, and specificity refers to PD detection. Dynamic HMM features included fraction occurrence, mean state duration, and transition probabilities. Static FC features were computed from Fisher *z*‐transformed Pearson correlations between Schaefer400 parcel wise BOLD time series, yielding 79,800 FC edges per participant.

Abbreviations: ACC, accuracy; AUC, area under the receiver operating characteristic curve; FC, functional connectivity.

### 3.6 Consistency of Functional States Across Parcellation, Clustering Schemes, and the Age‐Balanced Subset

We first assessed the consistency of functional brain states—particularly the unimodal state and the DAN&LIM state—across different parcellation schemes. Both states demonstrated stable topographical profiles when mapped to the Schaefer200 and Schaefer400 cortical atlases (Figure S5A). Correlation analyses of cluster centers between atlases further confirmed high spatial consistency (Schaefer200 vs. Schaefer400: *r* = 0.791, *p* < 0.001; *r* = 0.894, *p* < 0.001; Figure S5B). We next evaluated the stability of the unimodal state under varying clustering resolutions (*k* = 12–17). The state remained robust across different *k*‐values, as evidenced by high correlations in cluster centers (Figure S6A,B), indicating that its spatial configuration is largely invariant to clustering parameters.

To evaluate the potential influence of age disparities on our primary findings, we repeated the key analyses in an age‐balanced subset (*n* = 116), comprising 43 HC, 44 patients with PD, and 29 patients with PSP. Although the age imbalance was substantially reduced, the PSP group remained numerically older than the HC and PD groups, with pairwise *p* values of 0.08 and 0.09, respectively (demographic details in Table S4). In the independently refitted age‐balanced subset, candidate HMM solutions from *k* = 2 to *k* = 20 were evaluated. A topographically analogous unimodal state was identified across the *k* = 18–20 solutions shown in Figure S7. In the *k* = 19 solution, this state was labeled State 2 and was spatially analogous, rather than numerically identical, to State 15 in the full‐cohort *k* = 16 solution (Figure S7B). Notably, at *k* = 19, group differences in fraction occurrence of the unimodal state remained significant: PD patients showed reduced occupancy relative to HC (t = 2.05, *p* = 0.04), while PSP patients exhibited increased occupancy (t = −2.97, *p* < 0.01; Figure S8). Fraction occurrence of this state remained strongly different among groups after FDR correction (F = 12.17, *p* = 0.000016, pFDR = 0.00031). Post hoc comparisons showed higher fraction occurrence of the unimodal state (state 2 in the independently fitted age‐balanced *k* = 19 solution) in PSP than in PD (mean difference = 0.0347, *p* = 0.00026, pFDR = 0.0049). Additional FDR corrected fraction occurrence group effects were observed in state VIS&DMN, state DAN&FPCN, and state DAN, and pairwise analyses identified PSP‐related alterations in states FPCN, DAN, DAN&FPCN, as well as a PD‐related increase in State VIS&DMN (Table S5). These findings support the robustness of the principal unimodal‐state pattern after reducing the between‐group age imbalance, although residual age related confounding cannot be fully excluded.

## 4 Discussion

This study revealed striking, opposing alterations in the temporal dynamics of unimodal network‐dominant brain states in PD and PSP: while PD patients exhibited reduced fraction occurrence in this state, PSP patients showed a clear increase. Notably, these findings represent the first direct evidence that brain dynamic analysis can dissociate PD and PSP through a shared, quantifiable biomarker derived from the same functional state. Compared to healthy controls and PD, PSP patients presented additional abnormalities, including prolonged and more frequent occupancy of states predominated by dorsal attention and limbic networks (DAN&LIM), as well as overactivation within the dorsal attention and frontoparietal control network (DAN&FPCN). Furthermore, these temporal dynamics were closely linked to non‐motor symptom severity, underscoring their clinical relevance. Importantly, machine learning models leveraging these dynamic brain state features achieved better discrimination between PD and PSP than traditional functional connectivity measures.

Our observation of decreased occupancy and shortened durations in the unimodal visual‐sensorimotor state among PD patients aligns with previous studies indicating impaired integration and weaker functional interactions of visual and sensorimotor networks in PD [30], likely reflecting early breakdown of visuomotor integration central to motor control [31, 32]. Such dysfunction has been associated with greater reliance on visual feedback and altered gaze strategies, potentially contributing to motor deficits such as freezing of gait [33]. In contrast, PSP patients demonstrate increased duration and prevalence of unimodal network activity, which, according to emerging evidence [10, 12], may be attributed to progressive structural atrophy in the visual system and concomitant hyperconnectivity in secondary visual cortical regions [34]. This phenomenon could indicate compensatory or maladaptive cortical reorganization, as suggested by increased temporal synchrony and expanded intra‐cortical communication in response to ongoing structural decline [10, 12]. The distinct alterations observed in the unimodal network may therefore provide candidate imaging features for differentiating patients with established PD and PSP diagnoses, while also informing the pathophysiology underlying disrupted visual processing across parkinsonian syndromes.

Beyond the visual and sensorimotor domains, our results further establish that PSP patients experience prolonged occupancy within states dominated by executive and attentional control networks (DAN&LIM, DAN&FPCN), in line with prior findings of prominent frontal–parietal network dysfunction in PSP [16]. Alterations in the temporal landscape of these networks may result from underlying structural changes and network remodeling, ultimately contributing to impairments in cognitive flexibility and executive function that typify PSP.

Our analysis revealed that higher unimodal‐state fraction occurrence was associated with better cognitive performance in both PD and PSP. This finding suggests that flexibility within these networks is important for maintaining sensorimotor integration and visual–spatial attention, which support cognitive abilities. However, the association patterns with other non‐motor symptoms differ between the two groups. The clinical associations of unimodal state fraction occurrence were not unidirectional in PSP. Higher fraction occurrence was associated with better cognitive performance, as reflected by higher MoCA scores, but was also associated with greater non‐motor symptom burden, as reflected by higher MDS‐UPDRS‐I scores. These findings suggest that unimodal state dynamics may relate differently to distinct clinical domains rather than representing a simple marker of overall clinical improvement or deterioration. One possible explanation is that greater engagement of visual–sensorimotor networks may support cognitive performance while occurring alongside broader non‐motor impairment in PSP. However, this interpretation remains tentative, and the mixed pattern may also reflect clinical heterogeneity, residual confounding, or the limited PSP sample size. Separately, the positive association between DAN&LIM mean duration and ESS scores suggests that prolonged persistence of this executive–limbic state may be related to greater daytime sleepiness in PSP. In PD, this pattern was not evident, which may reflect fundamental mechanistic differences rooted in distinct neurodegenerative pathways. In PD, degeneration mainly affects dopaminergic and sensorimotor circuits, gradually reducing the adaptive capacity of these networks. PSP, on the other hand, is characterized by widespread tau pathology involving frontal and parietal regions, possibly leading to more severe mental and behavioral symptoms as well as persistent disturbances in network switching. These contrasting correlations between brain dynamics and clinical symptoms provide new insight into the different clinical manifestations of PD and PSP. The clinical correlation analyses further indicate that dynamic brain state metrics capture clinically meaningful heterogeneity within PSP. The most robust PSP‐specific finding was the positive association of global‐state fraction occurrence with both PSPRS and PSP‐CDS. This suggests that increased expression of a globally synchronized state may index more advanced or widespread network involvement in PSP. Our study supports the idea that distinct patterns of dynamic brain network dysfunction underlie the progression and symptom profiles of these two disorders. Moreover, our results show that dynamic brain state metrics achieved significantly better classification performance than traditional static connectivity measures. However, the class‐specific performance was imbalanced, with high specificity for PD (0.969) but only moderate sensitivity for PSP (0.637), corresponding to an average false‐negative proportion of approximately 36% for PSP across the outer cross‐validation folds. This imbalance is clinically relevant because PSP identification is often the more challenging diagnostic task. Therefore, the high overall AUC should not be interpreted as equivalent classification performance for both disorders or as evidence supporting stand‐alone clinical use. Moreover, because the classifier was developed and internally evaluated in participants with established clinical diagnoses, its performance cannot be assumed to generalize to patients with early or diagnostically uncertain parkinsonism. Rather, these HMM‐derived dynamic features should be regarded as an ancillary differential diagnostic aid that may complement clinical assessment. Before clinical application, the model requires validation in independent external multicenter cohorts, ideally in combination with structural MRI measures.

Several limitations should be acknowledged. First, although all patients were diagnosed by experienced movement disorder specialists according to internationally accepted clinical diagnostic criteria, which represents standard practice in clinical research, neuropathological confirmation or disease‐specific molecular imaging (e.g., tau or α‐synuclein imaging) were unavailable. Therefore, a small degree of diagnostic misclassification cannot be completely excluded and may have influenced the observed between‐group differences. Future studies incorporating molecular imaging or neuropathological confirmation are warranted to further validate these findings. Second, medication exposure represents an unresolved confound in the present study. Pre‐scan medication regimens were not standardized, and complete LEDD data and exact ON/OFF medication status were not systematically available. Because medication exposure and treatment responsiveness may differ between PD and PSP, these differences may have partly contributed to the observed group effects on resting‐state functional connectivity and network dynamics. Consequently, dopaminergic medication effects cannot be separated from disease‐related effects in the present analyses ([35, 36, 37, 38]). Third, while the original PSP cohort was significantly older than the PD and HC groups, we performed age‐balanced participant resampling from the PD and HC cohorts to align with the age distribution of the PSP group. All PSP participants were retained, whereas the youngest HC and PD participants were sequentially excluded. This procedure substantially reduced, but did not completely eliminate, the between‐group age differences. Moreover, because the HMM was independently refitted in the reduced subset rather than directly applying the original 16‐state model, the age‐balanced analysis should be interpreted as a complementary robustness assessment rather than an exact replication of the primary full‐cohort analysis. Residual age‐related confounding therefore cannot be fully excluded. Although gender was statistically controlled, residual confounding is possible. Furthermore, the PSP cohort was substantially smaller than the PD and HC groups, which may reduce statistical power for detecting small PSP‐related effects and increase uncertainty in effect‐size estimation. Sensitivity power analyses indicated that the current design was adequately powered to detect moderate‐to‐large group differences and correlations, while smaller effects may have been missed. Nevertheless, bootstrap analyses and confidence intervals supported the overall stability and directionality of the principal PSP‐specific associations. Fourth, structural MRI‐derived correction was not performed in the current study. Given the prominent structural neurodegeneration in PSP, regional atrophy may influence BOLD signal properties and dynamic functional metrics; therefore, some functional alterations may partly reflect underlying structural changes. In addition, although PSP phenotypic variants were recorded, subgroup‐specific analyses were not feasible due to limited sample sizes within individual phenotypes. Given the recognized clinical heterogeneity of PSP, future studies with larger subtype‐specific cohorts are needed to determine whether dynamic network alterations differ across PSP phenotypes. Therefore, these findings should still be interpreted cautiously and validated in larger multicenter cohorts; future work employing longitudinal designs and including larger, more diverse populations will be necessary. Lastly, the cross‐sectional nature of this study precludes definitive conclusions regarding disease progression and causality.

## 5 Conclusions

In summary, our findings provide novel insights into the temporal network dynamics differentiating PD and PSP, demonstrate associations between dynamic state properties and clinical symptom burden, and support the potential of dynamic brain‐state features as complementary imaging markers for differential diagnosis. We highlight that PD and PSP exhibit opposing dynamic profiles in unimodal and executive‐attentional networks that parallel mood and sleep disturbances. Future research should aim to further unravel disease‐specific dynamic network signatures and their mechanistic roles, thereby improving diagnostic precision and enabling more targeted interventions for atypical parkinsonian syndromes.

## Author Contributions

C.Y., J.M. and T.M. conceptualized this work. C.Y. and J.S. developed the analytical methods, analyzed the data, prepared the figures, and drafted the manuscript. J.F., T.W. and P.C. collected and co‐analyzed the data. J.S. preprocessed the imaging data.

## Funding

This study is supported by grants from the National Natural Science Foundation of China (62276081), National Key Research and Development Program (2025YFF0517803), Shenzhen Science and Technology Program (GXWD20231129121139001, JCYJ20240813110522029, JCYJ20250604145427037), Guangdong Basic and Applied Research Foundation (2023A1515010792, 2023B1515120065), China Initiative on Neurodegeneration and Aging (CHINA), and Guangdong S&T Programme 2025B0101130004.

## Ethics Statement

This study was performed in accordance with the Declaration of Helsinki and was approved by the Institutional Review Board of Xuanwu Hospital, with approval number: 2022‐047.

## Consent

Written informed consent was provided by all participants before being enrolled in this study.

## Conflicts of Interest

The authors declare no conflicts of interest.

## Supporting information


**Figure S1:** Correlation between brain states and the Yeo atlas networks. This figure shows the correlation between the cluster center of each state and the Yeo brain network atlas. Positive values indicate higher positive correlations. Asterisks denote significant associations between specific states and networks (*p* < 0.05). Different colors represent distinct brain networks. Notably, no significant network correlation was observed for State 2.
**Figure S2:** Transient brain states identified across all participants. (A) Cluster centers of the 16 brain states identified with the HMM using Schaefer400 parcels. (B) Visualization of cluster centers on the right lateral brain surface. (C) Visualization of cluster centers on the left lateral brain surface.
**Figure S3:** Frequency of occurrence in the state unimodal across different cluster counts. The frequency of occurrence in the state unimodal is shown for cluster counts ranging from *k* = 12 to *k* = 17. Similar results are observed across different clustering solutions, indicating robustness of the finding.
**Figure S4:** PSP‐specific clinical correlations with HMM dynamic metrics. Orange points indicate PSP participants. Annotated *r* values are covariate‐adjusted partial correlations; *q* values indicate FDR‐corrected *p* values within each scale‐by‐metric family.
**Figure S5:** Comparison of State unimodal and State DAN&LIM using different parcellation schemes. (A) Brain maps of the unimodal state and the DAN&LIM state identified using both Schaefer400 and Schaefer200 atlases. Red indicates positive activation; blue indicates functional inhibition. (B) Correlation of cluster centers for the same state across different atlases demonstrates high consistency (*r* = 0.791, *p* < 0.001 and *r* = 0.894, *p* < 0.001, respectively).
**Figure S6:** Stability of the unimodal state across different clustering numbers. (A) Brain maps of the unimodal state using the Schaefer400 atlas with cluster counts from *k* = 12 to *k* = 17. Red indicates positive activation; blue indicates functional inhibition. (B) The unimodal state remains stable across different cluster numbers, as shown by high correlation of cluster centers for the same state across different *k* values in the correlation matrix.
**Figure S7:** Sensitivity analysis of the state unimodal excluding age effects in 116 participants. (A) Correlation between the cluster centre of state unimodal with *k* = 19 in the age‐balanced subset and the Yeo brain network atlas. Positive values indicate higher positive correlations. Asterisks denote significant associations between specific states and networks (*p* < 0.05). Different colors represent distinct brain networks. (B) Brain maps of the unimodal state using the Schaefer400 atlas from *k* = 18 to *k* = 20. Red indicates positive activation; blue indicates functional inhibition.
**Figure S8:** Frequency of occurrence in state unimodal of *k* = 19 (*p < 0.05, and ***p < 0.005). Similar results are observed in *k* = 19 in the age‐balanced subset, indicating robustness of the finding.
**Table S1:** Fractional occupancy statistics across 16 HMM states.
**Table S2:** Mean dwell time statistics across 16 HMM states.
**Table S3:** PSP specific demographics and clinical dynamic metrics result.
**Table S4:** Demographic characteristics of remaining participants in three groups after age stratification and exclusion.
**Table S5:** Results of the age‐balanced sensitivity analysis.
**Table S6:** Sensitivity analyses of HMM‐derived differences between PD and PSP after adjustment for disease duration and clinical severity.
**Table S7:** State number for the principal HMM‐derived states reported in the main and sensitivity analyses.
**Table S8:** Complete targeted clinical correlations in PD and PSP.
**Table S9:** Complete PSP‐specific correlations of PSPRS and PSP‐CDS with HMM‐derived dynamic metrics.

## Data Availability

The data that support the findings of this study are available on request from the corresponding author. The data are not publicly available due to privacy or ethical restrictions. The codes for dynamic brain state analyses were conducted using the *hmmlearn* package (https://github.com/hmmlearn/hmmlearn) and modified from *pyleida* (https://psychomark.github.io/leida‐python/). Codes for machine learning used the *scikit‐learn* package (https://github.com/scikit‐learn/scikit‐learn). Sensitivity analyses used *neuromaps* (https://github.com/netneurolab/neuromaps). The complete codes are publicly available at https://github.com/Shijh123456/PD_PSP.
