# Supplementary material for: Unveiling the Distinctive Brain Functional Dynamics Between Parkinson's Disease and Progressive Supranuclear Palsy
Source: CNS Neurosci Ther. 2026 Sep 4;32(9):e71147. doi: 10.1002/cns.71147 (PMC13545542; doi:10.1002/cns.71147)
Supplement: Supplementary file 1 [file CNS-32-e71147-s001.docx]

## Supplementary Material

**Participants**

A total of 180 individuals were enrolled, including 69 healthy controls (HC; 30 males, 39 females), 82 patients with Parkinson disease (PD; 41 males, 41 females), and 29 patients with progressive supranuclear palsy (PSP; 19 males, 10 females). All participants meeting the study criteria were recruited from the Movement Disorders Clinic of Xuanwu Hospital, Capital Medical University. Patients with PD were diagnosed according to the Movement Disorder Society (MDS) clinical diagnostic criteria (Postuma et al., 2015), and patients with PSP were diagnosed according to the MDS criteria for PSP (Höglinger et al., 2017). Medication intake was not standardized before MRI acquisition. The inclusion criteria for the 69 healthy controls were as follows: (1) age over 40 years; (2) no history of neurological or psychiatric disorders; (3) no family history of movement disorders; (4) no significant brain abnormalities on structural magnetic resonance imaging (MRI). This study was approved by the Institutional Review Board of Xuanwu Hospital, Capital Medical University and written and informed consent was obtained from all participants before the study.

**Image acquisition.**

All participants were scanned using a 3T Siemens Magnetom Skyra MRI scanner. The scanner was equipped with a 20-channel head-neck combined coil receiver, with 16 channels used for scanning. Parameters of full-brain sagittal 3D T1-weighted magnetization-prepared rapid gradient-echo (MPRAGE) imaging were as follows: slice thickness = 1 mm; repetition time (TR) = 2530 ms; echo time (TE) = 2.98 ms; flip angle = 7°; display field of view (DFOV) = 256 * 256 * 192 pixels; voxel size = 1 * 1 * 1 mm³; scan time = 5 minutes 13 seconds. The 3D single-echo gradient-echo sequence parameters were as follows: slice thickness = 1.5 mm; TR = 25 ms; TE = 17.5 ms; echo = 1; flip angle = 15°; DFOV = 256 * 256 * 80 pixels; voxel size = 0.667 * 0.667 * 1.5 mm³; scan time = 5 minutes 06 seconds.

Resting-state fMRI data were acquired using an echo-planar imaging sequence sensitive to BOLD contrast. The exact EPI/BOLD acquisition parameters were as follows: TR = 2000 ms; TE = 30 ms; flip angle = 90°; field of view = 220x220 mm^2^; matrix size = 64 * 64; voxel size = 3.4 * 3.4 * 3 mm^3^; 35 axial slices with no gap, 176 repetitions; scan time = 5 minutes 52 seconds.

**Determining the number of HMM states to retain**

In HMM, fMRI time-series data are assumed to be representable as a sequence of a finite number of dynamic brain states (Whiteside, et al. 2021, Chu, et al. 2023). The number of states in HMM is an undefined parameter, which must be predetermined prior to model evaluation. In this study, we applied HMM with a range of states, from 2 to 18, to determine the optimal number of states that best characterizes the current data. This determination was based on several summary statistics. For each value of k, the clustering solution was assessed using the distortion index and the Davies-Bouldin index. The distortion index measures the degree of misfit in the clustering by evaluating the sum of squared distances between data points and their corresponding cluster centroids. Lower distortion values indicate better clustering performance, as they reflect a tighter grouping of data points within each state. The Davies-Bouldin index is a metric used to evaluate clustering quality by considering both the scatter within clusters and the separation between clusters. A lower Davies-Bouldin index suggests better clustering, as it indicates that the clusters are more distinct from one another and that the data points within each cluster are more cohesive. By adopting the “elbow” method, we considered the number 16 as the HMM parsing states in this study.

**Construction of the age-balanced subset**

All 29 participants with PSP were retained in the age-balanced sensitivity analysis. Participants in the HC and PD groups were ranked by age in ascending order, and the youngest individuals were sequentially excluded. Specifically, 26 of the original 69 HC participants and 38 of the original 82 participants with PD were excluded, resulting in final samples of 43 HC participants, 44 participants with PD, and 29 participants with PSP. Selection was based only on age and did not consider HMM-derived metrics, clinical outcomes, or group-difference results. No predefined age caliper was used. The procedure reduced but did not fully eliminate the age differences; therefore, the resulting sample is referred to as an age-balanced subset.

The HMM was refitted using the rs-fMRI data from these 116 participants. The original full-cohort HMM was not directly applied to or used for Viterbi decoding in the age-balanced subset. Candidate model orders were re-evaluated, and analogous states were identified according to their spatial network profiles. Candidate HMM model orders ranging from k = 2 to k = 20 were independently evaluated in the age-balanced subset. This wider search range was used in the reduced subset analysis and was distinct from the full-cohort search range of k = 2–18. An analogous unimodal state was observed in the k = 18–20 solutions, and the k = 19 solution was used for the detailed age-balanced group-comparison analysis. Because the age-balanced HMM was fitted independently, its state numbers were solution specific and were not assumed to correspond numerically to those of the full-cohort k = 16 solution.

**Supplementary Figure S1.** Correlation between brain states and the Yeo atlas networks. This figure shows the correlation between the cluster center of each state and the Yeo brain network atlas. Positive values indicate higher positive correlations. Asterisks denote significant associations between specific states and networks (p < 0.05). Different colors represent distinct brain networks. Notably, no significant network correlation was observed for State 2.

**Supplementary Figure S2.** Transient brain states identified across all participants. (A) Cluster centers of the sixteen brain states identified with the HMM using Schaefer400 parcels. (B) Visualization of cluster centers on the right lateral brain surface. (C) Visualization of cluster centers on the left lateral brain surface.

**Supplementary Figure S3.** Frequency of occurrence in the state unimodal across different cluster counts. The frequency of occurrence in the state unimodal is shown for cluster counts ranging from k = 12 to k = 17. Similar results are observed across different clustering solutions, indicating robustness of the finding.

**Supplementary Figure S4.** PSP-specific clinical correlations with HMM dynamic metrics. Orange points indicate PSP participants. Annotated r values are covariate-adjusted partial correlations; q values indicate FDR-corrected p values within each scale-by-metric family.

**Supplementary Figure S5.** Comparison of State unimodal and State DAN&LIM using different parcellation schemes. (A) Brain maps of the unimodal state and the DAN&LIM state identified using both Schaefer400 and Schaefer200 atlases. Red indicates positive activation; blue indicates functional inhibition. (B) Correlation of cluster centers for the same state across different atlases demonstrates high consistency (r = 0.791, p < 0.001 and r = 0.894, p < 0.001, respectively).

**Supplementary Figure S6.** Stability of the unimodal state across different clustering numbers. (A) Brain maps of the unimodal state using the Schaefer400 atlas with cluster counts from k = 12 to k = 17. Red indicates positive activation; blue indicates functional inhibition. (B) The unimodal state remains stable across different cluster numbers, as shown by high correlation of cluster centers for the same state across different k values in the correlation matrix.

**Supplementary Figure S7.** Sensitivity analysis of the state unimodal excluding age effects in 116 participants. (A) Correlation between the cluster centre of state unimodal with k = 19 in the age-balanced subset and the Yeo brain network atlas. Positive values indicate higher positive correlations. Asterisks denote significant associations between specific states and networks (p < 0.05). Different colours represent distinct brain networks. (B) Brain maps of the unimodal state using the Schaefer400 atlas from k = 18 to k = 20. Red indicates positive activation; blue indicates functional inhibition

**Supplementary Figure S8.** Frequency of occurrence in state unimodal of k = 19 (*indicates p < 0.05, and *** indicates p < 0.005). Similar results are observed in k = 19 in the age-balanced subset, indicating robustness of the finding.

**Supplementary Table S1.** Fractional occupancy statistics across 16 HMM states.

| **Omnibus group effect** | | | | | **Post-hoc pairwise contrasts** | | | | | | | | | | | | |
| --- | --- | --- | --- | --- | --- | --- | --- | --- | --- | --- | --- | --- | --- | --- | --- | --- | --- |
| **Label** | **F(group)** | **raw p** | **FDR p** | **PD vs HC** | | | | | **PSP vs HC** | | | | **PSP vs PD** | | | |  |
|  |  |  |  | **β** | | **95% CI** | **raw p** | **FDR p** | **β** | **95% CI** | **raw p** | **FDR p** | **β** | **95% CI** | **raw p** | **FDR p** |  |
| LIM | 0.169 | 0.845 | 0.926 | -0.003 | | [-0.012, 0.007] | 0.600 | 0.859 | 1.62e-04 | [-0.013, 0.014] | 0.981 | 0.983 | 0.003 | [-0.010, 0.016] | 0.687 | 0.785 |  |
| undefined | 2.311 | 0.102 | 0.217 | -0.010 | | [-0.025, 0.005] | 0.182 | 0.364 | -0.022 | [-0.043, -0.001] | 0.040 | 0.164 | -0.012 | [-0.032, 0.008] | 0.247 | 0.591 |  |
| VIS.A | 4.305 | 0.015 | 0.108 | 0.016 | | [0.005, 0.027] | 0.004 | 0.061 | 0.010 | [-0.006, 0.025] | 0.219 | 0.500 | -0.006 | [-0.021, 0.008] | 0.398 | 0.705 |  |
| SMN.A | 0.569 | 0.567 | 0.825 | 0.002 | | [-0.007, 0.012] | 0.630 | 0.859 | -0.005 | [-0.019, 0.009] | 0.492 | 0.970 | -0.007 | [-0.021, 0.006] | 0.292 | 0.591 |  |
| DMN.A | 1.209 | 0.301 | 0.535 | -0.007 | | [-0.017, 0.003] | 0.156 | 0.357 | 1.53e-04 | [-0.014, 0.014] | 0.983 | 0.983 | 0.007 | [-0.006, 0.021] | 0.296 | 0.591 |  |
| DAN | 2.252 | 0.108 | 0.217 | 0.009 | | [-0.001, 0.019] | 0.085 | 0.226 | -0.003 | [-0.018, 0.011] | 0.651 | 0.970 | -0.012 | [-0.026, 0.002] | 0.088 | 0.351 |  |
| VIS.B | 0.073 | 0.929 | 0.929 | -0.002 | | [-0.012, 0.008] | 0.710 | 0.859 | -4.62e-04 | [-0.015, 0.014] | 0.949 | 0.983 | 0.001 | [-0.012, 0.015] | 0.839 | 0.839 |  |
| DMN.B | 0.251 | 0.778 | 0.926 | -0.004 | | [-0.014, 0.007] | 0.480 | 0.853 | -0.002 | [-0.017, 0.012] | 0.772 | 0.970 | 0.002 | [-0.013, 0.016] | 0.831 | 0.839 |  |
| DAN&LIM | 1.022 | 0.362 | 0.579 | 6.18e-04 | | [-0.008, 0.009] | 0.889 | 0.889 | 0.009 | [-0.004, 0.021] | 0.174 | 0.464 | 0.008 | [-0.004, 0.020] | 0.195 | 0.591 |  |
| SMN.B | 3.689 | 0.027 | 0.108 | 0.014 | | [0.004, 0.024] | 0.008 | 0.062 | 0.010 | [-0.004, 0.025] | 0.172 | 0.464 | -0.004 | [-0.018, 0.010] | 0.595 | 0.756 |  |
| DMN.C | 0.141 | 0.869 | 0.926 | 0.002 | | [-0.008, 0.012] | 0.752 | 0.859 | -0.002 | [-0.016, 0.012] | 0.788 | 0.970 | -0.004 | [-0.018, 0.010] | 0.614 | 0.756 |  |
| global | 3.055 | 0.050 | 0.159 | -0.015 | | [-0.029, -6.32e-06] | 0.050 | 0.200 | -0.022 | [-0.043, -0.002] | 0.034 | 0.164 | -0.008 | [-0.028, 0.012] | 0.441 | 0.705 |  |
| transmodal | 0.145 | 0.865 | 0.926 | 8.22e-04 | | [-0.006, 0.008] | 0.819 | 0.874 | -0.002 | [-0.012, 0.008] | 0.719 | 0.970 | -0.003 | [-0.012, 0.007] | 0.593 | 0.756 |  |
| DAN&FPCN | 3.951 | 0.021 | 0.108 | -0.002 | | [-0.012, 0.008] | 0.699 | 0.859 | 0.018 | [0.003, 0.033] | 0.017 | 0.164 | 0.020 | [0.006, 0.034] | 0.006 | 0.051 |  |
| unimodal | 7.567 | 7.06e-04 | 0.011 | -0.012 | | [-0.022, -0.001] | 0.026 | 0.141 | 0.016 | [6.51e-04,0.031] | 0.041 | 0.164 | 0.028 | [0.013, 0.042] | 2.56e-04 | 0.004 |  |
| DMN.D | 2.503 | 0.085 | 0.217 | 0.009 | | [-7.45e-04, 0.019] | 0.069 | 0.221 | -0.003 | [-0.018, 0.011] | 0.634 | 0.970 | -0.013 | [-0.027, 0.001] | 0.072 | 0.351 |  |

Note: Omnibus statistics were obtained from covariate-adjusted GLMs including group, age, sex, and mean FD. Post-hoc pairwise contrasts were estimated within the same model framework. β values indicate adjusted group differences between the first-listed and second-listed groups. Raw p-values and 95% confidence intervals are shown for each contrast. FDR p-values were corrected across the 16 HMM states within the metric and contrast. Highlighted rows indicate the principal corrected or supportive/exploratory states discussed in the main text.

**Supplementary Table S2.** Mean dwell time statistics across 16 HMM states.

| **Omnibus group effect** | | | | | **Post-hoc pairwise contrasts** | | | | | | | | | | | |  |
| --- | --- | --- | --- | --- | --- | --- | --- | --- | --- | --- | --- | --- | --- | --- | --- | --- | --- |
| **Label** | **F(group)** | **raw p** | **FDR p** | **PD vs HC** | | | | | **PSP vs HC** | | | | **PSP vs PD** | | | | |
|  |  |  |  | **β** | | **95% CI** | **raw p** | **FDR p** | **β** | **95% CI** | **raw p** | **FDR p** | **β** | **95% CI** | **raw p** | **FDR p** | |
| LIM | 1.634 | 0.198 | 0.396 | 0.161 | | [-0.316, 0.639] | 0.505 | 0.849 | 0.622 | [-0.057, 1.302] | 0.072 | 0.352 | 0.461 | [-0.200, 1.122] | 0.171 | 0.546 | |
| undefined | 1.774 | 0.173 | 0.395 | -0.830 | | [-1.724, 0.064] | 0.069 | 0.365 | -0.718 | [-1.991, 0.555] | 0.267 | 0.459 | 0.112 | [-1.127, 1.351] | 0.858 | 0.915 | |
| VIS.A | 2.712 | 0.069 | 0.239 | 0.515 | | [0.035, 0.994] | 0.036 | 0.284 | 0.594 | [-0.089, 1.276] | 0.088 | 0.352 | 0.079 | [-0.586, 0.743] | 0.815 | 0.915 | |
| SMN.A | 0.176 | 0.839 | 0.895 | 0.073 | | [-0.258, 0.404] | 0.663 | 0.885 | -0.048 | [-0.519, 0.423] | 0.841 | 0.897 | -0.121 | [-0.579, 0.337] | 0.603 | 0.821 | |
| DMN.A | 4.401 | 0.014 | 0.219 | -0.332 | | [-0.781, 0.117] | 0.147 | 0.546 | 0.588 | [-0.052, 1.227] | 0.072 | 0.352 | 0.919 | [0.297, 1.542] | 0.004 | 0.064 | |
| DAN | 3.001 | 0.052 | 0.239 | 0.387 | | [-0.168, 0.943] | 0.171 | 0.546 | -0.534 | [-1.326, 0.257] | 0.184 | 0.459 | -0.922 | [-1.692, -0.152] | 0.019 | 0.129 | |
| VIS.B | 0.105 | 0.900 | 0.900 | -0.055 | | [-0.642, 0.533] | 0.854 | 0.976 | 0.134 | [-0.703, 0.970] | 0.753 | 0.860 | 0.189 | [-0.626, 1.003] | 0.648 | 0.821 | |
| DMN.B | 0.284 | 0.753 | 0.894 | -0.148 | | [-0.860, 0.564] | 0.682 | 0.885 | -0.381 | [-1.396, 0.633] | 0.459 | 0.565 | -0.233 | [-1.220, 0.754] | 0.641 | 0.821 | |
| DAN&LIM | 2.444 | 0.090 | 0.239 | -0.113 | | [-0.731, 0.505] | 0.719 | 0.885 | 0.834 | [-0.046, 1.714] | 0.043 | 0.352 | 0.946 | [0.090, 1.803] | 0.031 | 0.129 | |
| SMN.B | 0.668 | 0.514 | 0.748 | 0.237 | | [-0.204, 0.677] | 0.290 | 0.581 | 0.265 | [-0.362, 0.892] | 0.405 | 0.565 | 0.029 | [-0.581, 0.639] | 0.926 | 0.926 | |
| DMN.C | 1.306 | 0.274 | 0.486 | -0.217 | | [-0.605, 0.170] | 0.270 | 0.581 | -0.424 | [-0.976, 0.128] | 0.131 | 0.421 | -0.207 | [-0.744, 0.331] | 0.449 | 0.821 | |
| global | 2.614 | 0.076 | 0.239 | -1.020 | | [-1.908, -0.131] | 0.025 | 0.284 | -0.751 | [-2.016, 0.515] | 0.243 | 0.459 | 0.269 | [-0.962, 1.500] | 0.667 | 0.821 | |
| transmodal | 0.246 | 0.782 | 0.894 | 0.224 | | [-0.480, 0.929] | 0.531 | 0.849 | -0.023 | [-1.026, 0.981] | 0.965 | 0.965 | -0.247 | [-1.223, 0.730] | 0.619 | 0.821 | |
| DAN&FPCN | 0.393 | 0.675 | 0.894 | -0.018 | | [-0.427, 0.391] | 0.931 | 0.992 | 0.230 | [-0.352, 0.812] | 0.437 | 0.565 | 0.248 | [-0.319, 0.815] | 0.389 | 0.821 | |
| unimodal | 2.477 | 0.087 | 0.239 | -0.331 | | [-0.869, 0.208] | 0.227 | 0.581 | 0.485 | [-0.282, 1.252] | 0.214 | 0.459 | 0.816 | [0.069, 1.562] | 0.032 | 0.129 | |
| DMN.D | 0.678 | 0.509 | 0.748 | 0.003 | | [-0.646, 0.653] | 0.992 | 0.992 | -0.501 | [-1.425, 0.424] | 0.287 | 0.459 | -0.504 | [-1.404, 0.395] | 0.270 | 0.721 | |

Note: Omnibus statistics were obtained from covariate-adjusted GLMs including group, age, sex, and mean FD. Post-hoc pairwise contrasts were estimated within the same model framework. β values indicate adjusted group differences between the first-listed and second-listed groups. Raw p-values and 95% confidence intervals are shown for each contrast. FDR p-values were corrected across the 16 HMM states within the metric and contrast. Highlighted rows indicate the principal corrected or supportive/exploratory states discussed in the main text.

**Supplementary Table S3.** PSP specific demographics and clinical dynamic metrics result

| **Clinical scale** | **Dynamic metric** | **n** | **partial r** | **raw p** | **p_fdr** | **Bootstrap 95% CI** | **Bootstrap p** |
| --- | --- | --- | --- | --- | --- | --- | --- |
| PSPRS | Global FO | 24 | 0.758 | <0.001 | <0.001 | 0.487 to 0.923 | <0.001 |
| PSP-CDS | Global FO | 24 | 0.655 | <0.001 | 0.008 | 0.285 to 0.880 | 0.004 |
| PSP-CDS | DAN&FPCN FO | 24 | -0.481 | 0.0274 | 0.1463 | −0.772 to −0.049 | 0.033 |

**Supplementary Table S4**. Demographic characteristics of remaining participants in three groups after age stratification and exclusion

|  | HC | PD | PSP | p (HC vs. PD) | p (HC vs. PSP) | p (PD vs. PSP) |
| --- | --- | --- | --- | --- | --- | --- |
| age | 64.72±6.05 | 64.61±5.53 | 67.24±5.56 | 0.93 | 0.08 | 0.09 |

Note: Participants with age imbalance across groups were excluded to minimize age-related confounding. Data for age are presented as mean ± standard deviation. P-values were calculated using independent-samples t-tests to compare age differences between each pair of groups. HC, Healthy Control; PD, patients with Parkinson’s disease; PSP, patients with progressive supranuclear palsy.

**Supplementary Table S5.** Results of the age-balanced sensitivity analysis

| **Metric** | **State** | **F** | **raw p** | **FDR q** |
| --- | --- | --- | --- | --- |
| FO | unimodal | 12.171 | 1.63e-05 | 0.00031 |
| FO | VIS&DMN | 6.048 | 0.0032 | 0.0304 |
| FO | DAN&FPCN | 5.288 | 0.00638 | 0.0404 |
| FO | DAN | 4.808 | 0.00991 | 0.0471 |

**Supplementary Table S6.** Sensitivity analyses of HMM-derived differences between PD and PSP after adjustment for disease duration and clinical severity.

| Dynamic metric | Model | β (PSP−PD) | 95% CI | p | qFDR |
| --- | --- | --- | --- | --- | --- |
| Unimodal FO | Base | 0.0256 | 0.0109 to 0.0402 | <0.001 | 0.0125 |
| Unimodal FO | + Disease duration | 0.0269 | 0.0107 to 0.0431 | 0.0014 | 0.0226 |
| Unimodal FO | + MDS-UPDRS-III | 0.0241 | 0.0080 to 0.0402 | 0.0037 | 0.0584 |
| Unimodal FO | + MDS-UPDRS-I | 0.0186 | 0.0034 to 0.0337 | 0.0168 | 0.2691 |
| DAN&FPCN FO | Base | 0.0162 | 0.0011 to 0.0313 | 0.0355 | 0.2842 |
| DAN&FPCN FO | + Disease duration | 0.0158 | −0.0008 to 0.0323 | 0.0614 | 0.4097 |
| DAN&FPCN FO | + MDS-UPDRS-III | 0.0123 | −0.0037 to 0.0283 | 0.1300 | 0.5821 |
| DAN&FPCN FO | + MDS-UPDRS-I | 0.0126 | −0.0036 to 0.0288 | 0.1259 | 0.5036 |
| DAN&LIM dwell time | Base | 0.7020 | −0.1745 to 1.5784 | 0.1153 | 0.4612 |
| DAN&LIM dwell time | + Disease duration | 0.7778 | −0.1834 to 1.7391 | 0.1115 | 0.5948 |
| DAN&LIM dwell time | + MDS-UPDRS-III | 0.6909 | −0.2767 to 1.6585 | 0.1596 | 0.6334 |
| DAN&LIM dwell time | + MDS-UPDRS-I | 0.7296 | −0.2500 to 1.7092 | 0.1426 | 0.6182 |

Note: Analyses were restricted to participants with PD and PSP. The base model included diagnosis, age, sex, and mean framewise displacement. Disease duration, MDS-UPDRS-III, or MDS-UPDRS-I was additionally included as indicated. Disease duration was defined as the interval between reported symptom onset and MRI acquisition and was expressed in years. Positive β values indicate higher values in PSP than in PD. FDR correction was performed across the 16 states separately within each dynamic-metric family and model. FO, fractional occupancy; DAN, dorsal attention network; FPCN, frontoparietal control network; LIM, limbic network.

**Supplementary Table S7.** State number for the principal HMM-derived states reported in the main and sensitivity analyses.

| Analysis | HMM solution | State number | State label |
| --- | --- | --- | --- |
| Primary full cohort analysis | k = 16 | State 9 | DAN&LIM |
| Primary full cohort analysis | k = 16 | State 14 | DAN&FPCN |
| Primary full cohort analysis | k = 16 | State 15 | Unimodal |
| Age balanced sensitivity analysis | k = 19 | State 2 | Unimodal |

Note: State numbers are specific to each independently fitted HMM solution and therefore are not directly comparable across solutions. The unimodal pattern corresponded to state 15 in the primary 16-state solution and state 2 in the 19-state solution derived from the age-balanced subset. State labels were assigned according to their dominant canonical network profiles. DAN, dorsal attention network; FPCN, frontoparietal control network; LIM, limbic network.

**Supplementary Table S8.** Complete targeted clinical correlations in PD and PSP

| **Group** | **Clinical measure** | **HMM-derived metric** | **n** | **Partial r** | **95% CI** | **Raw p** | **qFDR** |
| --- | --- | --- | --- | --- | --- | --- | --- |
| PD | MDS-UPDRS-I | Unimodal FO | 78 | -0.047 | -0.271 to 0.182 | 0.6882 | 0.8156 |
| PD | MDS-UPDRS-I | DAN&FPCN FO | 78 | -0.027 | -0.253 to 0.201 | 0.8156 | 0.8156 |
| PD | MDS-UPDRS-I | DAN&LIM mean dwell time | 78 | 0.160 | -0.069 to 0.374 | 0.1695 | 0.5084 |
| PD | MDS-UPDRS-II | Unimodal FO | 78 | -0.015 | -0.241 to 0.213 | 0.9001 | 0.9001 |
| PD | MDS-UPDRS-II | DAN&FPCN FO | 78 | -0.123 | -0.341 to 0.107 | 0.2929 | 0.8786 |
| PD | MDS-UPDRS-II | DAN&LIM mean dwell time | 78 | 0.019 | -0.208 to 0.245 | 0.8683 | 0.9001 |
| PD | MDS-UPDRS-III | Unimodal FO | 78 | 0.051 | -0.178 to 0.275 | 0.6629 | 0.7195 |
| PD | MDS-UPDRS-III | DAN&FPCN FO | 78 | -0.116 | -0.334 to 0.114 | 0.3203 | 0.7195 |
| PD | MDS-UPDRS-III | DAN&LIM mean dwell time | 78 | 0.042 | -0.187 to 0.267 | 0.7195 | 0.7195 |
| **PD** | **MoCA** | **Unimodal FO** | **77** | **0.262** | **0.021 to 0.451** | **0.0233** | **0.0496** |
| PD | MoCA | DAN&FPCN FO | 77 | -0.000 | -0.229 to 0.228 | 0.9999 | 0.9999 |
| **PD** | **MoCA** | **DAN&LIM mean dwell time** | **77** | **0.255** | **0.028 to 0.457** | **0.0282** | **0.0496** |
| PD | NMSS | Unimodal FO | 72 | -0.074 | -0.305 to 0.166 | 0.5470 | 0.9917 |
| PD | NMSS | DAN&FPCN FO | 72 | -0.031 | -0.266 to 0.207 | 0.7994 | 0.9917 |
| PD | NMSS | DAN&LIM mean dwell time | 72 | -0.001 | -0.238 to 0.235 | 0.9917 | 0.9917 |
| PD | RBDQ-HK | Unimodal FO | 78 | 0.001 | -0.226 to 0.228 | 0.9952 | 0.9952 |
| PD | RBDQ-HK | DAN&FPCN FO | 78 | 0.051 | -0.178 to 0.275 | 0.6646 | 0.9952 |
| **PD** | **RBDQ-HK** | **DAN&LIM mean dwell time** | **78** | **0.285** | **0.062 to 0.481** | **0.0132** | **0.0395** |
| PD | ESS | Unimodal FO | 73 | -0.062 | -0.293 to 0.176 | 0.6108 | 0.6108 |
| PD | ESS | DAN&FPCN FO | 73 | 0.078 | -0.160 to 0.308 | 0.5192 | 0.6108 |
| PD | ESS | DAN&LIM mean dwell time | 73 | 0.114 | -0.125 to 0.340 | 0.3489 | 0.6108 |
| **PSP** | **MDS-UPDRS-I** | **Unimodal FO** | **26** | **0.439** | **0.062 to 0.745** | **0.0312** | **0.0804** |
| PSP | MDS-UPDRS-I | DAN&FPCN FO | 26 | -0.314 | -0.643 to 0.112 | 0.1440 | 0.2161 |
| PSP | MDS-UPDRS-I | DAN&LIM mean dwell time | 26 | -0.071 | -0.469 to 0.352 | 0.7490 | 0.7490 |
| PSP | MDS-UPDRS-II | Unimodal FO | 25 | 0.132 | -0.306 to 0.525 | 0.5571 | 0.7482 |
| PSP | MDS-UPDRS-II | DAN&FPCN FO | 25 | -0.471 | -0.010 to 0.700 | 0.0560 | 0.1681 |
| PSP | MDS-UPDRS-II | DAN&LIM mean dwell time | 25 | -0.073 | -0.480 to 0.360 | 0.7482 | 0.7482 |
| PSP | MDS-UPDRS-III | Unimodal FO | 25 | -0.089 | -0.492 to 0.346 | 0.6951 | 0.8099 |
| PSP | MDS-UPDRS-III | DAN&FPCN FO | 25 | -0.342 | -0.667 to 0.094 | 0.1198 | 0.3594 |
| PSP | MDS-UPDRS-III | DAN&LIM mean dwell time | 25 | -0.054 | -0.465 to 0.376 | 0.8099 | 0.8099 |
| **PSP** | **MoCA** | **Unimodal FO** | **20** | **0.570** | **0.122 to 0.824** | **0.0130** | **0.0511** |
| PSP | MoCA | DAN&FPCN FO | 20 | 0.459 | -0.028 to 0.770 | 0.0638 | 0.0958 |
| PSP | MoCA | DAN&LIM mean dwell time | 20 | 0.311 | -0.199 to 0.689 | 0.2240 | 0.2240 |
| PSP | NMSS | Unimodal FO | 20 | 0.017 | -0.468 to 0.494 | 0.9486 | 0.9486 |
| PSP | NMSS | DAN&FPCN FO | 20 | -0.526 | -0.804 to -0.061 | 0.0300 | 0.0899 |
| PSP | NMSS | DAN&LIM mean dwell time | 20 | 0.097 | -0.403 to 0.552 | 0.7124 | 0.9486 |
| PSP | RBDQ-HK | Unimodal FO | 22 | 0.145 | -0.331 to 0.562 | 0.5538 | 0.7909 |
| PSP | RBDQ-HK | DAN&FPCN FO | 22 | 0.065 | -0.401 to 0.504 | 0.7909 | 0.7909 |
| PSP | RBDQ-HK | DAN&LIM mean dwell time | 22 | 0.357 | -0.116 to 0.698 | 0.1330 | 0.3989 |
| PSP | ESS | Unimodal FO | 24 | 0.362 | -0.083 to 0.686 | 0.1073 | 0.1609 |
| PSP | ESS | DAN&FPCN FO | 24 | -0.139 | -0.538 to 0.312 | 0.5488 | 0.5488 |
| **PSP** | **ESS** | **DAN&LIM mean dwell time** | **24** | **0.559** | **0.176 to 0.801** | **0.0067** | **0.0228** |

**Supplementary Table S9.** Complete PSP-specific correlations of PSPRS and PSP-CDS with HMM-derived dynamic metrics

| **Clinical scale** | **HMM-derived metric** | **n** | **Partial r** | **Raw p** | **qFDR** | **Bootstrap 95% CI** | **Bootstrap p** |
| --- | --- | --- | --- | --- | --- | --- | --- |
| PSPRS | LIM FO | 24 | 0.141 | 0.5422 | 0.7880 | -0.433 to 0.572 | 0.6483 |
| **PSPRS** | **Undefined FO** | **24** | **0.621** | **0.0026** | **0.0212** | **0.115 to 0.838** | **0.0200** |
| PSPRS | VIS.A FO | 24 | -0.365 | 0.1034 | 0.4139 | -0.628 to 0.001 | 0.0506 |
| PSPRS | SMN.A FO | 24 | -0.026 | 0.9100 | 0.9100 | -0.455 to 0.406 | 0.9215 |
| PSPRS | DMN.A FO | 24 | 0.135 | 0.5582 | 0.7880 | -0.254 to 0.586 | 0.4650 |
| PSPRS | DAN FO | 24 | -0.203 | 0.3763 | 0.7880 | -0.558 to 0.293 | 0.3572 |
| PSPRS | VIS.B FO | 24 | 0.160 | 0.4896 | 0.7880 | -0.265 to 0.566 | 0.3958 |
| PSPRS | DMN.B FO | 24 | -0.201 | 0.3816 | 0.7880 | -0.576 to 0.271 | 0.3890 |
| PSPRS | DAN&LIM FO | 24 | -0.119 | 0.6078 | 0.7880 | -0.485 to 0.355 | 0.4836 |
| PSPRS | SMN.B FO | 24 | -0.119 | 0.6060 | 0.7880 | -0.514 to 0.536 | 0.8043 |
| PSPRS | DMN.C FO | 24 | -0.035 | 0.8802 | 0.9100 | -0.514 to 0.486 | 0.8365 |
| **PSPRS** | **Global FO** | **24** | **0.758** | **<0.0001** | **0.0003** | **0.492 to 0.922** | **0.0002** |
| PSPRS | Transmodal FO | 24 | 0.032 | 0.8903 | 0.9100 | -0.519 to 0.496 | 0.9135 |
| PSPRS | DAN&FPCN FO | 24 | -0.365 | 0.1035 | 0.4139 | -0.722 to 0.146 | 0.1514 |
| PSPRS | Unimodal FO | 24 | -0.153 | 0.5086 | 0.7880 | -0.561 to 0.258 | 0.4152 |
| PSPRS | DMN.D FO | 24 | -0.108 | 0.6403 | 0.7880 | -0.495 to 0.434 | 0.6905 |
| PSPRS | LIM mean dwell time | 24 | 0.169 | 0.4643 | 0.9945 | -0.270 to 0.574 | 0.4414 |
| PSPRS | Undefined mean dwell time | 24 | 0.092 | 0.6910 | 0.9945 | -0.283 to 0.459 | 0.6013 |
| PSPRS | VIS.A mean dwell time | 24 | -0.435 | 0.0487 | 0.3898 | -0.776 to -0.011 | 0.0450 |
| PSPRS | SMN.A mean dwell time | 24 | 0.300 | 0.1859 | 0.9180 | -0.052 to 0.637 | 0.0758 |
| PSPRS | DMN.A mean dwell time | 24 | -0.033 | 0.8883 | 0.9945 | -0.481 to 0.535 | 0.9997 |
| PSPRS | DAN mean dwell time | 24 | 0.022 | 0.9240 | 0.9945 | -0.421 to 0.501 | 0.9783 |
| PSPRS | VIS.B mean dwell time | 24 | 0.023 | 0.9211 | 0.9945 | -0.612 to 0.524 | 0.8935 |
| PSPRS | DMN.B mean dwell time | 24 | -0.034 | 0.8827 | 0.9945 | -0.416 to 0.395 | 0.8673 |
| PSPRS | DAN&LIM mean dwell time | 24 | -0.241 | 0.2924 | 0.9180 | -0.610 to 0.204 | 0.2540 |
| PSPRS | SMN.B mean dwell time | 24 | -0.084 | 0.7174 | 0.9945 | -0.519 to 0.323 | 0.7069 |
| PSPRS | DMN.C mean dwell time | 24 | -0.002 | 0.9945 | 0.9945 | -0.417 to 0.459 | 0.9293 |
| PSPRS | Global mean dwell time | 24 | 0.230 | 0.3159 | 0.9180 | -0.043 to 0.469 | 0.0824 |
| PSPRS | Transmodal mean dwell time | 24 | 0.217 | 0.3443 | 0.9180 | -0.204 to 0.604 | 0.2558 |
| PSPRS | DAN&FPCN mean dwell time | 24 | -0.484 | 0.0262 | 0.3898 | -0.781 to -0.044 | 0.0322 |
| PSPRS | Unimodal mean dwell time | 24 | -0.113 | 0.6255 | 0.9945 | -0.594 to 0.288 | 0.5211 |
| PSPRS | DMN.D mean dwell time | 24 | 0.019 | 0.9348 | 0.9945 | -0.408 to 0.428 | 0.9493 |
| PSP-CDS | LIM FO | 24 | 0.008 | 0.9713 | 0.9713 | -0.577 to 0.520 | 0.9699 |
| PSP-CDS | Undefined FO | 24 | 0.545 | 0.0107 | 0.0853 | 0.066 to 0.790 | 0.0284 |
| PSP-CDS | VIS.A FO | 24 | -0.237 | 0.3015 | 0.7398 | -0.535 to 0.114 | 0.1586 |
| PSP-CDS | SMN.A FO | 24 | 0.199 | 0.3862 | 0.7398 | -0.158 to 0.538 | 0.2442 |
| PSP-CDS | DMN.A FO | 24 | 0.187 | 0.4161 | 0.7398 | -0.189 to 0.638 | 0.3130 |
| PSP-CDS | DAN FO | 24 | -0.095 | 0.6830 | 0.9390 | -0.433 to 0.387 | 0.6495 |
| PSP-CDS | VIS.B FO | 24 | 0.089 | 0.7026 | 0.9390 | -0.342 to 0.474 | 0.5955 |
| PSP-CDS | DMN.B FO | 24 | -0.279 | 0.2208 | 0.7241 | -0.613 to 0.189 | 0.2184 |
| PSP-CDS | DAN&LIM FO | 24 | -0.192 | 0.4035 | 0.7398 | -0.568 to 0.292 | 0.3388 |
| PSP-CDS | SMN.B FO | 24 | 0.013 | 0.9546 | 0.9713 | -0.381 to 0.564 | 0.8419 |
| PSP-CDS | DMN.C FO | 24 | -0.036 | 0.8774 | 0.9713 | -0.478 to 0.432 | 0.8607 |
| **PSP-CDS** | **Global FO** | **24** | **0.655** | **0.0013** | **0.008** | **0.279 to 0.880** | **0.0046** |
| PSP-CDS | Transmodal FO | 24 | -0.088 | 0.7043 | 0.9390 | -0.531 to 0.280 | 0.6475 |
| PSP-CDS | DAN&FPCN FO | 24 | -0.481 | 0.0274 | 0.1463 | -0.772 to -0.049 | 0.0336 |
| PSP-CDS | Unimodal FO | 24 | -0.276 | 0.2263 | 0.7241 | -0.666 to 0.139 | 0.1536 |
| PSP-CDS | DMN.D FO | 24 | 0.040 | 0.8626 | 0.9713 | -0.293 to 0.554 | 0.7753 |
| PSP-CDS | LIM mean dwell time | 24 | 0.085 | 0.7136 | 0.9321 | -0.391 to 0.517 | 0.7369 |
| PSP-CDS | Undefined mean dwell time | 24 | 0.054 | 0.8156 | 0.9321 | -0.369 to 0.503 | 0.7839 |
| PSP-CDS | VIS.A mean dwell time | 24 | -0.394 | 0.0769 | 0.3141 | -0.739 to 0.019 | 0.0576 |
| PSP-CDS | SMN.A mean dwell time | 24 | 0.429 | 0.0522 | 0.3141 | 0.109 to 0.742 | 0.0184 |
| PSP-CDS | DMN.A mean dwell time | 24 | -0.002 | 0.9922 | 0.9922 | -0.432 to 0.535 | 0.9385 |
| PSP-CDS | DAN mean dwell time | 24 | 0.091 | 0.6957 | 0.9321 | -0.359 to 0.564 | 0.7159 |
| PSP-CDS | VIS.B mean dwell time | 24 | 0.138 | 0.5514 | 0.9321 | -0.509 to 0.570 | 0.6731 |
| PSP-CDS | DMN.B mean dwell time | 24 | -0.172 | 0.4568 | 0.9321 | -0.555 to 0.247 | 0.3694 |
| PSP-CDS | DAN&LIM mean dwell time | 24 | -0.424 | 0.0552 | 0.3141 | -0.725 to -0.023 | 0.0396 |
| PSP-CDS | SMN.B mean dwell time | 24 | -0.083 | 0.7219 | 0.9321 | -0.508 to 0.248 | 0.5813 |
| PSP-CDS | DMN.C mean dwell time | 24 | -0.025 | 0.9140 | 0.9749 | -0.431 to 0.430 | 0.9583 |
| PSP-CDS | Global mean dwell time | 24 | 0.068 | 0.7705 | 0.9321 | -0.283 to 0.366 | 0.6397 |
| PSP-CDS | Transmodal mean dwell time | 24 | 0.099 | 0.6683 | 0.9321 | -0.273 to 0.498 | 0.5273 |
| PSP-CDS | DAN&FPCN mean dwell time | 24 | -0.392 | 0.0785 | 0.3141 | -0.693 to 0.030 | 0.0652 |
| PSP-CDS | Unimodal mean dwell time | 24 | -0.122 | 0.5977 | 0.9321 | -0.560 to 0.221 | 0.4120 |
| PSP-CDS | DMN.D mean dwell time | 24 | 0.068 | 0.7706 | 0.9321 | -0.313 to 0.532 | 0.6361 |

**Supplementary References**

1. Postuma RB, Berg D, Stern M, Poewe W, Olanow CW, Oertel W, et al. MDS clinical diagnostic criteria for Parkinson's disease. Movement Disorders. 2015;30(12):1591-1601.
2. Höglinger GU, Respondek G, Stamelou M, Kurz C, Josephs KA, Lang AE, et al. Clinical diagnosis of progressive supranuclear palsy: The Movement Disorder Society criteria. Movement Disorders. 2017;32(6):853-864.
3. Whiteside DJ, Jones PS, Ghosh BCP, Coyle-Gilchrist I, Gerhard A, Hu MT, et al. Altered network stability in progressive supranuclear palsy. Neurobiol Aging. 2021;107:109-17.
4. Chu C, Liu S, He N, Zeng Z, Wang J, Zhang Z, et al. Subthalamic stimulation modulates motor network in Parkinson's disease: recover, relieve and remodel. Brain. 2023;146(7):2780-91.
